# Rapid prediction of full spin systems using uncertainty-aware machine learning†

**DOI:** 10.1039/d3sc01930f

**Published:** 2023-09-15

**Authors:** Jake Williams, Eric Jonas

**Affiliations:** a Department of Computer Science, University of Chicago Chicago USA williamsjl@uchicago.edu ericj@uchicago.edu

## Abstract

Accurate simulation of solution NMR spectra requires knowledge of all chemical shift and scalar coupling parameters, traditionally accomplished by heuristic-based techniques or *ab initio* computational chemistry methods. Here we present a novel machine learning technique which combines uncertainty-aware deep learning with rapid estimates of conformational geometries to generate Full Spin System Predictions with UnCertainty (FullSSPrUCe). We improve on previous state of the art in accuracy on chemical shift values, predicting protons to within 0.209 ppm and carbons to within 1.213 ppm. Further, we are able to predict all scalar coupling values, unlike previous GNN models, achieving ^3^*J*_HH_ accuracies between 0.838 Hz and 1.392 Hz on small experimental datasets. Our uncertainty quantification shows a strong, useful correlation with accuracy, with the most confident predictions having significantly reduced error, including our top-80% most confident proton shift predictions having an average error of only 0.140 ppm. We also properly handle stereoisomerism and intelligently augment experimental data with *ab initio* data through disagreement regularization to account for deficiencies in training data.

## Abstract 2

FullSSPrUCe is an uncertainty-aware deep learning system which predicts all spin system parameters from 2D structures through rapid estimates of conformational geometries.

## Graphical abstract

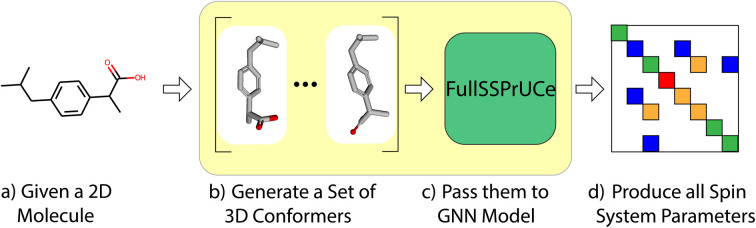

## 1 Introduction

Nuclear magnetic resonance (NMR) spectroscopy is a non-destructive spectroscopic tool for the understanding and elucidation of molecular structure. Due to the challenges in procuring and analyzing experimental results, recent work has focused on computational generation of spectral parameters to help chemists. Computational results can be used to confirm experimental analysis^1^ or as rapid estimations for downstream tasks such as stereoisomer identification.^2^

Computational methods for generating, predicting and understanding NMR parameters are studied and implemented at multiple levels of theory through the use of a variety of physics-based and machine learning techniques. Towards the higher levels of theory are first principle (*ab initio*) techniques, which use our knowledge of physics and chemistry to compute molecular properties. A frequently used *ab initio* technique is density functional theory (DFT), which models electron densities to calculate molecular properties. Advances in DFT have allowed it to produce high quality shift and coupling values^3,4^ which allows them to be used in a variety of practical applications and as a common point of comparison.^1,5,6^ Towards the lower levels of theory are machine learning (ML) techniques, which primarily use data to determine their predictions. We explore the advancements in ML models in Section 2.

One of the major distinctions between techniques and individual models is their use of a 2D or 3D representation of a molecule. Molecules are commonly represented as graphs for the purposes of instruction and modeling, and this extends to some computational techniques. The mathematical representation of a graph, a set of vertices and the edges which connect them, is a two dimensional structure. Molecules cannot be described solely by their connectivity, however, as 3D properties such as varying bond angles and lengths can greatly impact the shape of a molecule and its resulting spectrum. This occurs due to the relatively long acquisition period of NMR (usually between 50 ms and up to a few second),^7^ allowing the device to observe multiple conformations of the same molecule and the spectrum to be composed of their averages.^8^ It is also susceptible to isomeric differences,^2^ which are not observed in 2D structures.

DFT (along with most *ab initio* techniques) uses exact atom coordinates rather than a graph. Machine learning techniques, on the other hand, vary greatly in their usage of 2D or 3D structures. Those that do use 3D structures typically do so by generating a conformer or set of conformers using DFT.^9,10^ DFT is slow, often prohibitively so, thus other methods for generating conformers may be considered, including minimizing force fields, parallel-tempering and distance geometry techniques, each with their own approximation to speed tradeoff. ETKDG^11^ is one such distance geometry technique which incorporates experimental and physical knowledge to improve its conformer generation.

Using machine learning and the 3D structure of a molecule, we seek to predict all chemical shift and coupling parameters, and provide a measure of uncertainty in our predictions. In this paper, we will briefly explore other machine learning based techniques for NMR prediction to highlight our improvements. We will then demonstrate our success across multiple prediction tasks and performance metrics, before giving a detailed explanation of our methodology.

## 2 Prior work

NMR parameter prediction by learning a model through data dates back to the 1970s and the introduction of HOSE codes.^12^ This early work used featurization of atomic neighborhoods with data to create nearest neighbor models for predicting unknown spectra. Investments into larger datasets and machine learning techniques have given rise to new methods, such as IMPRESSION,^9^ which uses Kernel Ridge Regression to predict shift and coupling values. These methods both seek to categorize the local environment of an atom and then use well established mathematical techniques to compare the environment to those in a dataset. However, HOSE codes are generated from a 2D structure, while IMPRESSION requires a 3D structure obtained *via* DFT as input.^13^

With the advancement of deep learning, other recent models have adopted graph neural networks (GNNs) for this prediction task. Graph neural networks operate on a graph (a set of vertices and the edges that connect them) to predict per-vertex, per-edge, and per-graph properties. This connects naturally to the graphical model of a molecule and GNNs enforce relational biases^14^ that should be highly advantageous in NMR prediction tasks. Some networks, such as the model produced by Jonas and Kuhn,^15^ use a 2D molecule representation to strictly fit the GNN paradigm. Others, such as the CASCADE model,^10^ use the full 3D structure from DFT to better inform predictions. The 2D *versus* 3D representation questions may have limited some of these applications, as GNN models thus far have been limited solely to predicting chemical shifts, with no predictions of scalar couplings.

The further these models move from *ab initio* based techniques, the more important it is to understand their modes of failure. Even DFT can have major breakdowns that make it unreliable in some domains.^16^ Understanding whether a prediction can be trusted and to what degree is an important direction of work that is rarely addressed in machine learning tasks. Previous works have used ensembling^9^ or ensembling-like^15^ techniques to measure the uncertainty in their predictions, and we follow in a similar vein.

Our model generates Full Spin System Predictions with UnCertainty (FullSSPrUCe), meaning it predicts both chemical shift and scalar coupling values using a graph neural network with a 3D structure. Further, where previous ML methods which use 3D structures have taken their structures from DFT, we use distance geometries from ETKDG to make faster predictions. Lastly, we continue the previous work done to model uncertainty by providing a quantified estimate with each prediction. Thus, our model improves on the state-of-the-art by making more accurate predictions of full spin systems using conformers generated with faster methods, while providing a quantified estimate of uncertainty.

## 3 Results

**Fig. 1:**
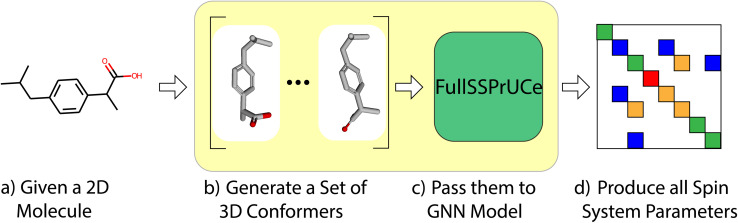
In the standard prediction task, we take as input the 2D representation of any small molecule (a). We then generate a set of 3D conformers (b) which serve as input to our GNN based model (c). This allows us to generate the spin system parameters that fully determine the molecule's spectrum (d).

First, we compare the accuracy of our model to previous works by examining its chemical shift and scalar coupling values on a well-established dataset. As shown in Fig. 1, this is a straight forward task in which we take each molecule in the dataset, generate its conformers, and then make predictions using our model. We then compare to the ground truth from the dataset to produce a mean average error (MAE) across the dataset. We compare our MAE to the other models on experimental and *ab initio* results. For more details on the structure of the model (the “FullSSPrUCe” box in Fig. 1), see Section 4.

We will then consider the impact of our uncertainty quantification as a tool for sorting and selecting predictions. We perform uncertainty quantification by showing different portions of our model different subsets of the training data. We can then use all sections of the model at test time and compare their differences to estimate uncertainty, as shown in Fig. 9. As opposed to traditional ensemble methods, such as IMPRESSION,^9^ the result is a single model, trained only once, but with many of the benefits of full ensemble techniques. We refer to this method as bootstrapping, alluding to a similar method in statistics. This method is explained in detail in Section 4.4.

We then look at the impact of using ETKDG for conformer generation. We explore both the differences in performance and time for our model. We will also consider the downstream task of stereoisomer identification, which is enabled by the 3D structures and inspired by DP4.^2^

Lastly, we consider a prediction task in which both experimental and *ab initio* training data is available. This has traditionally been attempted using transfer learning,^10^ however we introduce a new method we call disagreement regularization. Disagreement regularization learns to predict experimental and *ab initio* data simultaneously, moderating the loss function along two channels by how similar the experimental and *ab initio* ground truth values are. See Section 4.5 for details on this loss function.

### 3.1 FullSSPrUCe accurately predicts shifts and coupling

We compare initially to the other machine learning based models IMPRESSION,^9^ CASCADE,^10^ and the Jonas and Kuhn GNN.^15^ IMPRESSION is a kernel-based technique, which compares the local environments of atoms based on DFT conformers. CASCADE is a set of three GNN based models, one for predicting *ab initio* values from DFT-optimized geometries (DFTNN), one for predicting experimental values from DFT-optimized geometries (ExpNN-dft), and one for predicting experimental values from molecular mechanics based geometries (ExpNN-ff). Jonas and Kuhn's GNN uses 2D structures to predict experimental values.

Throughout this section, each of our models is trained and evaluated on data from the NMRShiftDB.^17^ This is a user-contributed database of experimental NMR shift values for small molecules, which has been used previously to train and evaluate machine learning models, including those to which we are comparing our model.^10,15^ For our purposes, we further narrowed down the molecules to use to those with at most 128 atoms (including protons) and with only atoms H, C, O, N, F, S, P and Cl, resulting in approximately 33k molecules (see ESI† for more details). For each of these molecules, which have existing experimental parameters, we used DFT to compute an additional set of parameters. Thus, for the NMRShiftDB molecules, we have two ways that we can train and evaluate models – on experimental or *ab initio* data. Note that in both cases, the input remains the same – a 2D representation (SMILES string, *etc.*) of a molecule, from which we will use ETKDG to generate conformers. This means that when performing an *ab initio* prediction task, we are not predicting the results from the same final conformer that the DFT method would have. Here, models trained on experimental data are evaluated on experimental data and those trained on *ab initio* data are evaluated on *ab initio*, and we will explore mixing these types in later sections.

**Fig. 2:**
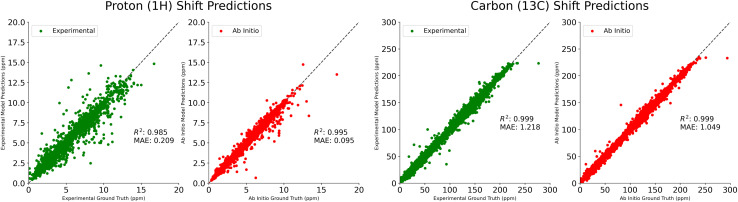
Comparison of predicted proton (^1^H) and carbon (^13^C) shifts to true labels from experimental (green) and *ab initio* (red) testing datasets, using models trained and evaluated on experimental and *ab initio* NMRShiftDB datasets, respectively. All shifts reported in ppm.

In Table 1, we compare our mean average error on ^1^H and ^13^C shift prediction tasks. We train four models using the NMRShiftDB data described above, split according to shift type (protons *vs.* carbons) and experimental *vs. ab initio*. Jonas and Kuhn uses this same dataset, but only the experimental data. IMPRESSION performs only *ab initio* prediction, having done their own DFT calculations on molecules from the Cambridge Structural Database,^18^ using adaptive sampling to obtain a training set of 882 molecules. CASCADE does both experimental and *ab initio* predictions, performing their own DFT calculations on an 8 K molecule subset of NMRShiftDB, and using a further 5 K subset of ^13^C experimental data that they believe is reliable. FullSSPrUCe provides a clear and significant improvement over the comparison models on all four prediction tasks, including 5.0% and 16.7% improvement over CASCADE on *ab initio*^1^H and ^13^C, respectively. Scatter plots of our predictions *versus* the ground truth for these four tasks are provided in Fig. 2.

**Table 1:** Comparison of chemical shift testing accuracy, as measured by the mean average error (MAE) in ppm, to other ML models. Where available, we compare proton (^1^H) and carbon (^13^C) predictions on both experimental and *ab initio* NMRShiftDB data

Model	Proton experimental	Proton *ab initio*	Carbon experimental	Carbon *ab initio*
Gerrard:IMPRESSION^9^	—	0.260	—	2.310
Guan:CASCADE^10^	—	0.100	1.250(dft), 1.430(ff)	1.260
GNN (Jonas and Kuhn^15^)	0.280	—	1.430	—
FullSSPrUCe	0.209 ± 0.005	0.095 ± 0.002	1.218 ± 0.008	1.049 ± 0.013

**Fig. 3:**
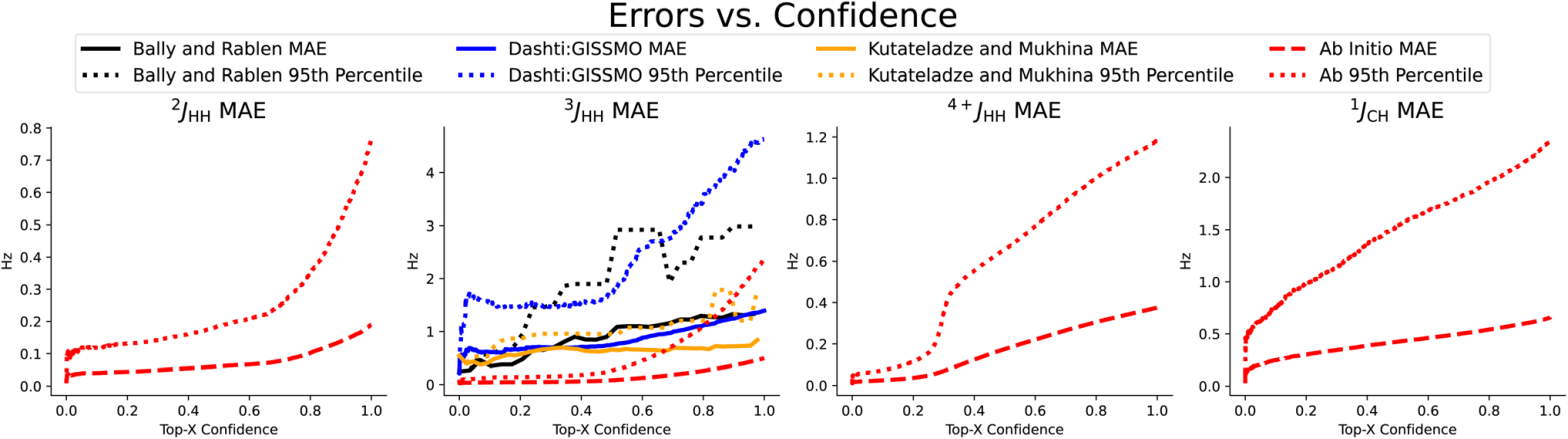
Coupling prediction accuracy on previously chosen subset of coupling types, measured as the rolling mean average error and rolling 95th percentile error in Hz as quantified uncertainty increases. We observe a strong correlation, even on the three small experimental datasets shown here,^4,19,20^ again demonstrating the usefulness of the quantified uncertainty in selecting for the predictions with the best accuracy.

In Table 2, we compare our mean average error on a subset of scalar couplings. Here we trained and tested only on *ab initio* data, because we did not have any large enough experimental datasets. We used this *ab initio* trained model to examine our performance on three small experimental datasets in Fig. 3. Note that CASCADE and Jonas and Kuhn's GNN do not make any coupling predictions and IMPRESSION predicted only ^1^*J*_CH_ coupling, where we made on average 22.0% more accurate predictions.

**Table 2:** Measurements of coupling testing accuracy, as measured by the mean average error (MAE) in Hz, for ^1^*J*_CH_, ^2^*J*_HH_, ^3^*J*_HH_, and ^4^*J*_HH_ couplings. Models trained and evaluated on *ab initio* NMRShiftDB data only, due to lack of availability of experimental coupling values for training

Model	^1^ *J*_CH_	^2^ *J*_HH_	^3^ *J*_HH_	^4^ *J*_HH_
Gerrard:IMPRESSION^9^	0.870	—	—	—
FullSSPrUCe	0.679 ± 0.014	0.194 ± 0.005	0.504 ± 0.011	0.121 ± 0.002

### 3.2 Uncertainty quantification allows better understanding of predictions

**Fig. 4:**
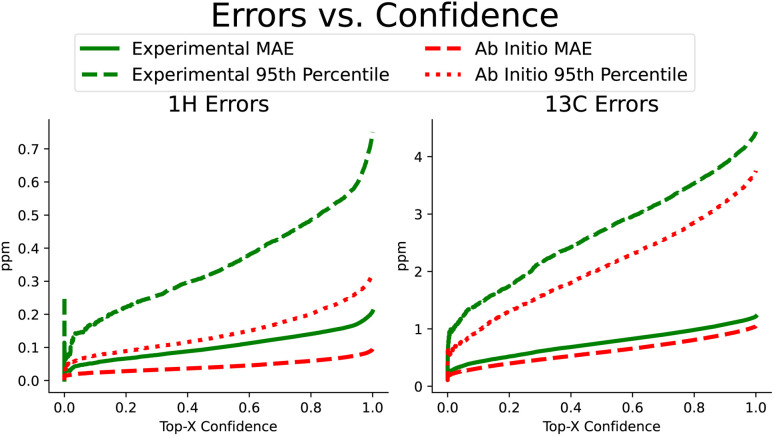
Proton and carbon chemical shift prediction accuracy, measured as the rolling mean average error and rolling 95th percentile error in ppm as quantified uncertainty increases. These plots demonstrate the connection between the quantified uncertainty and prediction error, allowing a user to potentially select for the top most confident predictions and expect them to have significantly lower error.

Beyond improvements in accuracy, FullSSPrUCe also provides a quantified estimate of uncertainty, pairing each prediction with a value intended to help a user understand the expected accuracy of a prediction (see Section 4.4). Here, we use the same NMRShiftDB data as in the previous section, including keeping all experimental and *ab initio* data separate. In Fig. 4, we have sorted each prediction on a single atom by its corresponding uncertainty. We then plot the rolling mean average error and the rolling 95th percentile error as uncertainty increases, demonstrating the correlation between our uncertainty and accuracy. Further, this connection is present in both experimental and *ab initio* data, which is extremely useful when we know that there are different reasons for noisy data in these two domains (*e.g.* misassignments in experimental data *versus* approximations in *ab initio* data). By comparing the experimental MAEs to CASCADE, we see that our methods provide a 3.2% improvement over ExpNN-dft and 15.4% over ExpNN-ff, the more similar model based on how we generate geometric features. However, this ability to sort our data according to confidence can increase this performance gap if we care only about a top percentage of predictions. Such uncertainty values are often used to identify, and potentially remove, likely errors. IMPRESSION used a variance cutoff to remove molecules with a high likelihood of incorrect labeling, demonstrating that it consistently removed outliers.^9^ Removing just 20% of the predictions that we are least confident in, our proton and carbon experimental mean average errors improve significantly, moving to 0.140 ppm and 0.981 ppm, respectively.

We repeat this procedure for the subset of scalar coupling values we addressed earlier in Fig. 3. Recall that we only performed training on *ab initio* data due to the lack of experimental data. We did collect a small amount of experimental coupling data from three sources,^4,19,20^ though these were used only as additional test sets for our ^3^*J*_HH_ accuracy. We again see the strong correlation between increased uncertainty and increased error. On *ab initio* coupling predictions, taking only the top-80% most confident predictions improves MAE performance between 14.4 and 47.7%. A measure of uncertainty can be crucial in understanding and utilizing predictions. Our uncertainty measure is simple to train and effective at test time in identifying predictions' expected accuracy.

This sorted, rolling error measurement provides a useful way to visualize the relationship between error and the uncertainty quantification. However, we may also want a numerical estimate of this relationship, so we directly measure the correlation between the error and the uncertainty values for each model, as reported in Table 3. We see that the correlation is meaningfully positive for all models, including when evaluating the coupling models on the selected small external datasets.

**Table 3:** Correlation between error and uncertainty quantification value for each prediction by the given models

Dataset	Parameter	Correlation
NMRShiftDB experimental	^1^H	0.496
	^13^C	0.309
NMRShiftDB *ab initio*	^1^H	0.499
	^13^C	0.305
	^2^ *J*_HH_	0.455
	^3^ *J*_HH_	0.512
	^4^ *J*_HH_	0.487
	^1^ *J*_CH_	0.297
Bally and Rablen^4^	^3^ *J*_HH_	0.375
Dashti:GISSMO^19^	^3^ *J*_HH_	0.405
Kutateladze and Mukhina^20^	^3^ *J*_HH_	0.496

### 3.3 Examining the effects of 3D structures

Next, we will consider the effects of using the 3D structure of a molecule as defined by an ensemble of conformers on predictions of full spin system parameters. First, we will look at the tradeoff between the number of conformers in the ensemble. Then we will look at a downstream task known as stereoisomer identification, which is enabled by the use of 3D structures.

#### 3.3.1 Distance geometry conformers speed up predictions without significant performance loss

**Fig. 5:**
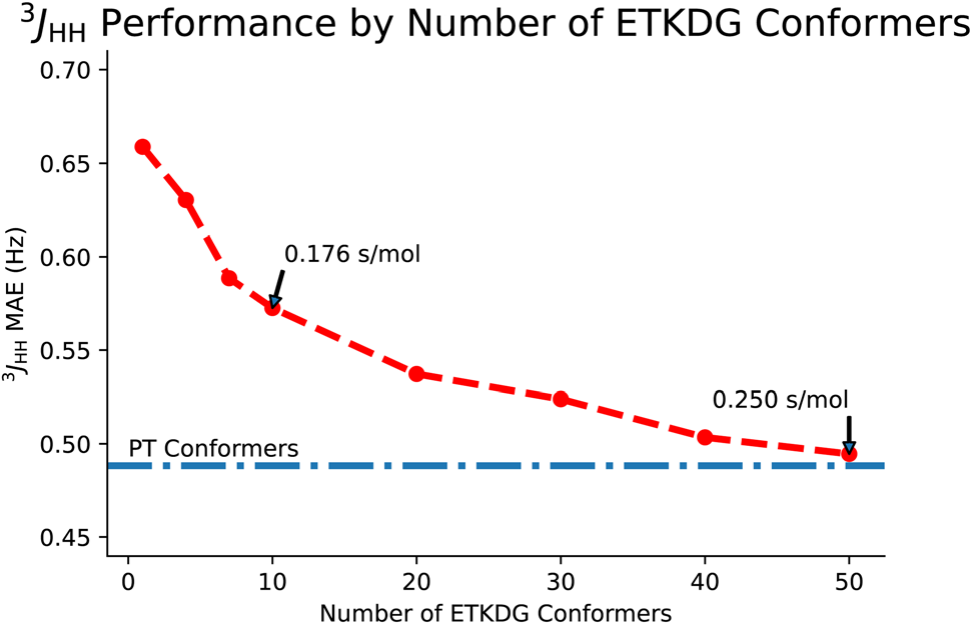
A comparison of FullSSPrUCe performance on *ab initio*^3^*J*_HH_ scalar couplings, as measured by mean average error in Hz, with different numbers of ETKDG conformers used to generate features. For reference, the performance using conformers generated from parallel-tempering (PT) is provided, demonstrating that enough ETKDG conformers can nearly recover PT performance, but fewer conformers still achieve strong performance while taking less time to generate.

We improved greatly on both experimental and *ab initio* prediction tasks by using an ensemble of ETKDG^11^ conformers to generate features for our GNN. ETKDG draws rapid samples from an implicit distribution over conformers, so generating more samples, while slower, better represents the space of conformers. After generating the conformers, we apply a single MMFF94 optimization step to each, obtaining their Boltzmann weights, and use these weights to combine the features from each conformer in the ensemble. In Fig. 5, we plot our ^3^*J*_HH_ coupling mean average error as a function of the number of ETKDG conformers used to generate the features for each model. These models were trained using the NMRShiftDB *ab initio* data discussed in Section 3.1. We also include a model with features taken from an ensemble of conformers generated using parallel-tempering. Compared to a single, much slower DFT conformer (as used by CASCADE and IMPRESSION), our ensembles of conformers perform quite well, with large ensembles allowing us to surpass previous performance. In fact, enough ETKDG conformers are sufficient to nearly replicate performance with our own higher quality conformer sets (PT). We also observe a tradeoff between number of conformers and performance that appears to have an asymptotic approach to top-end performance.

Given the similarities in performance, the main motivation for using ETKDG is that distance geometry techniques are faster. In Table 4, we examine the difference in time taken to generate an ensemble of conformers across multiple methods. We also note that each of these methods are approximately linear in time to generate larger sets of conformers. Returning to Fig. 5, the tradeoff between accuracy and number of conformers can be interpreted as accuracy and time taken. However, the tradeoff is not strictly linear here and so we could pick a point along the curve which balances the tradeoff to a desired level for our application. In this paper, we use 50 conformers to demonstrate the top end accuracy of our models, but for other tasks (such as predicting datasets on the order of tens of millions of molecules), using an ensemble with only 10 conformers may be appropriate.

**Table 4:** Comparison of time (single-core) taken to generate an ensemble of conformers for each of 10 K molecules, and the time taken for FullSSPrUCe to make full spin system predictions for each molecule

Method	Time taken per molecule
DFT	2 days
PT	1 h
ETKDG	0.176 s
FullSSPrUCe	0.064 s

**Fig. 6:**
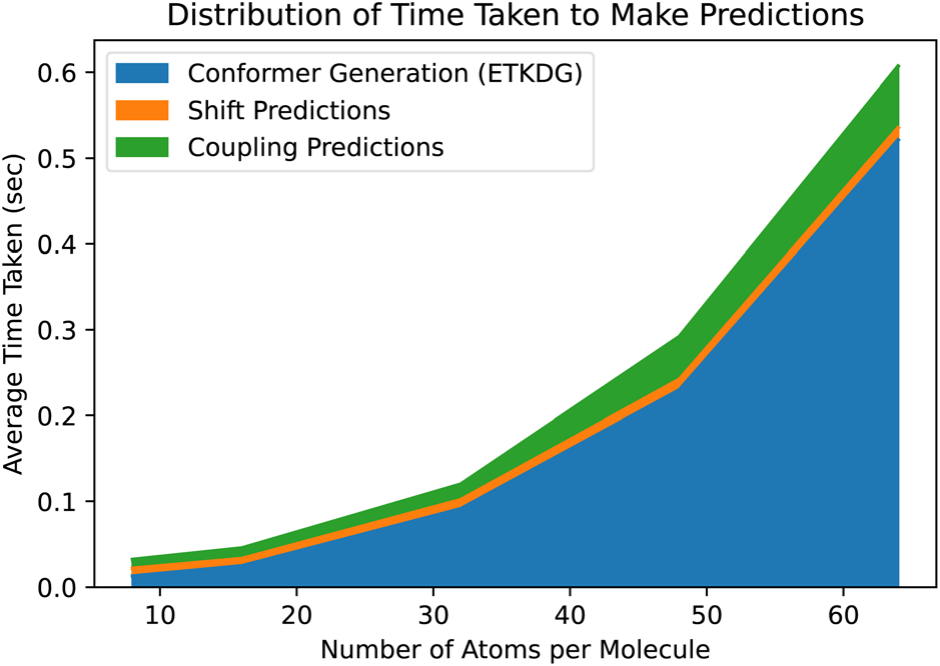
The prediction pipeline consists of three parts: generating ETKDG conformers, predicting shift values and predicting coupling values. Even with a fast method such as ETKDG, generating the conformers takes the majority of the time, and scales poorly with the number of atoms in the molecules.

While ETKDG is faster than either PT or DFT, and the conformers it generates are good enough to allow our models to achieve similar accuracy, it is still the bottleneck operation in the prediction pipeline, as shown in Fig. 6. This will become even more of an issue as we investigate larger, more diverse molecules as the time taken to generate conformers for larger molecules scales quadratically. However, the features that get passed to the model represent summary statistics of the set of conformers generated. This means that lower quality and fewer conformers matter less in general as we have shown so far. There still may be room for molecular geometry featurization to be sped up further, whether through other methods for generating conformers or skipping that step entirely, without losing significant model accuracy.

#### 3.3.2 3D structures enable stereoisomer identification

Stereoisomers, which have identical graph topology but different spatial properties, and therefore different physical properties, provide an interesting test of the usefulness of our 3D features and how they enhance our predictions. The geometric features we generate from our conformer ensembles allow us to make different predictions for different stereoisomers of the same molecule, where features that see exclusively graph topology cannot. These predictions are of particular interest for their usefulness in downstream tasks, such as stereoisomer identification using the DP4 probability function.

For this task, we use a new dataset, specifically a subset of GDB-17.^21^ GDB-17 contains multiple different stereoisomers of many of the molecules in its dataset. Using DFT, we can then obtain separate NMR parameters for each stereoisomer. We selected molecules using the same criteria as in Section 3.1, resulting in a dataset with approximately 66k total molecules (see ESI† for more details). We retrain our model for predicting ^1^H and ^13^C shifts on this dataset, in the hopes that a model which has seen only data with many stereoisomers of the same molecules will be useful for the purpose of performing stereoisomer identification. We base our work on stereoisomer identification on the work in creating the DP4 probability.^2^ In the original problem set up, the experimental ^1^H and ^13^C shift parameters are known for a molecule, but the particular stereoisomer is unknown. The goal is to identify which of a set of possible stereoisomers the experimental parameters were obtained from. In the original work, Smith and Goodman generated *ab initio* calculations of the parameters for each stereoisomer, and compared them to the experimentally derived parameters to generate a distribution over the set of stereoisomers. Here, we take the *ab initio* data in GDB-17 as the known parameters, and use our model to generate comparison parameters for the set of possible stereoisomers to identify the most likely candidate.

For each of 980 test molecules, we generated up to 7 other stereoisomers (if there were enough permutations available), for a total of 8 candidate structures. This resulted in 665 of the molecules having 8 candidate structures, and the majority of the remaining molecules having 4 candidate structures. We then use FullSSPrUCe to generate predicted ^1^H and ^13^C shift values and DP4 to assign probabilities to each candidate structure. We are able to correctly identify the true structure approximately 49% of the time, including 45% of the time when a molecule has 8 candidate structures, and achieve top-2 accuracy on 73% of molecules (65% for 8 candidate molecules). Further breakdowns of the results are provided in the ESI.†

Our model clearly outperforms random guesses by a significant margin, but it is not as accurate as we might have expected. When training FullSSPrUCe on GDB-17, we cannot use all structures in the database due to its enormous size (∼166 billion molecules), and so we may not get a representative sample when choosing our subset to train on. In fact, we observe a degradation in testing performance of almost twice as much error (0.162 ppm for protons and 2.117 ppm for carbons). This work shows that our model has the capacity to distinguish between stereoisomers using proton and carbon shifts, but needs to be trained and tested more thoroughly before it can be consistently relied upon for these decisions in the same manner that we use DFT.

### 3.4 Disagreement regularization intelligently combines experimental and *ab initio* training data

Finally, we consider the task of training from experimental and *ab initio* data simultaneously, and so we turn to our disagreement regularization loss. This task is particularly interesting and difficult because of the differences between experimental and *ab initio* data. Note that going forward, we will leave behind the GDB-17 data and return to using exclusively the NMRShiftDB data, for which we have both experimental and *ab initio* parameters. We would like to predict experimental data as accurately as possible, but it has a host of issues: experimental error such as misassignments, failure to distinguish diastereomers, experimental variability such as unreported solvents, and general noisiness. *Ab initio* data on the other hand is perfectly replicable and noiseless, however it has its own sources of error, as discussed earlier. There are also differences between expense and ease of generation of data. Experimental data can be very difficult to obtain, especially high quality data or for rare samples and materials. *Ab initio* data, though sometimes slow, can be obtained for any substance through simulation.

These difficulties have been acknowledged in previous works. IMPRESSION and Jonas and Kuhn each chose to work exclusively with either *ab initio* or experimental data due in part to these differences. CASCADE, on the other hand, used both to create three models (the DFTNN, ExpNN-dft and ExpNN-ff models previous discussed). They began by training DFTNN on 8 K molecules with *ab initio* assignments, then used transfer learning to train to experimental assignments. However, it is important to note that to train their experimental models, they used only a 5 K molecule subset of their total training dataset, removing the molecules where experimental data and *ab initio* data disagreed beyond a certain threshold. Transfer learning provides a way to incorporate *ab initio* data into the training process, while still prioritizing experimental data, but it does not address the issues we have discussed in experimental data.

We attempt to address the strengths and weaknesses of both types of data through our new technique of disagreement regularization. We train jointly on both types of data, using a model with two output channels (one for each type of data), but that share the majority of their parameters. The loss function then prioritizes accuracy on the experimental channel, but compares the ground truth on each channel to determine how much to do so (see full details in Section 4.5). We evaluated this technique by dividing NMRShiftDB^17^ into two unique subsets.

We split the data into two subsets based on the presence of ‘small rings’ in the molecule, namely those rings with exactly 3 or 4 atoms. These rings create unique geometric and chemical properties, and so we expect that making predictions about NMR properties on these molecules without training data regarding them will be difficult. However, we can easily obtain *ab initio* data for these molecules to see how disagreement regularization helps us recover our performance. For this experiment, we focused only on proton shift prediction.

Our results are summarized in Table 5. We begin with two models trained strictly on experimental data: the baseline and the experimental control. The baseline is our normal experimental model, trained with experimental data from all ‘areas’ of chemical space that we are interested in. It shows that small ring molecules tend to be slightly more difficult to predict than big ring molecules, averaging 0.247 ppm error, as opposed to the big ring MAE of 0.205 ppm. The experimental control is a model trained with only experimental big ring data, so that it has never seen any molecules with small rings. It has a nearly identical big ring MAE, but its small ring MAE increases significantly to 1.248 ppm. This demonstrates that small rings operate as an area of chemical space that requires training data to predict accurately. However, as we know, we cannot always get experimental data from all areas of chemical space. Our goal then is to construct a model which performs well on the experimental small rings without having seen any such experimental training data.

**Table 5:** We trained four models on different subsets of NMRShiftDB data, with only the final model being trained using disagreement regularization. We compare their performance on ^1^H shifts for atoms in molecules with rings of 3 or 4 atoms

Model	Experimental data?		*Ab initio* data?		MAE (ppm)	
	Big rings	Small rings	Big rings	Small rings	Big rings	Small rings
Baseline	✓	✓			0.205	0.247
Experimental control	✓				0.206	1.248
*Ab initio* control	✓			✓	0.205	0.283
Disagreement (*λ* = 5)	✓		✓	✓	0.205	0.268
Disagreement (*λ* = 10)	✓		✓	✓	0.243	0.206

We start with a naive implementation of this, which is to add in all of the *ab initio* data from the small rings that we have and train jointly. This is referred to as the *ab initio* control model. Adding in the *ab initio* small ring data does not impact the big ring MAE significantly, however it does bring the small ring MAE back to a reasonable value at 0.283 ppm. This is still worse than the baseline, which is to be expected given that we know *ab initio* data has systematic error when evaluated on experimental data. Therefore, training directly to *ab initio* data (including models trained with only *ab initio* data) will not be able to recover the baseline performance.

**Fig. 7:**
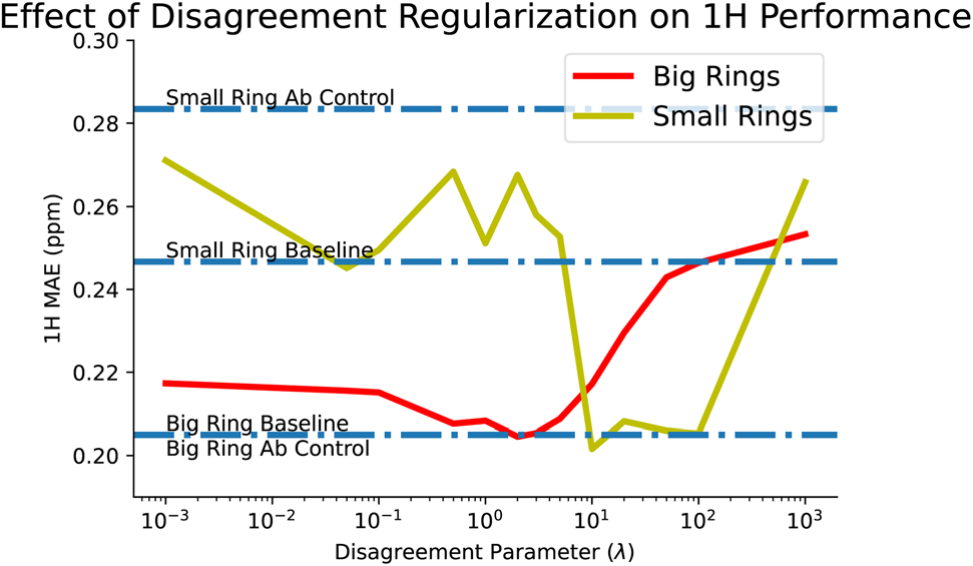
Disagreement regularization balances the loss between *ab initio* and experimental predictions through the hyperparameter *λ*, allowing it to learn better predictions in unseen experimental areas of space (small rings), while also maintaining or even improving on predictions in seen areas (big rings). Optimal performance is to not replicate either experimental or *ab initio* data exactly by finding a middle ground for *λ*, though unfortunately the best *λ*s for each of big and small rings do not exactly overlap.

This brings us to our disagreement regularization models, which we designed in the hopes of tackling these sorts of tasks. In this case, we provide all of the *ab initio* data that we have, for both big and small rings, along with the experimental big rings. Using disagreement regularization, we are able to recover the big ring baseline while also reducing small ring error to 0.268 ppm, a significant improvement over the controls. We also explored the effect of our tuning parameter, *λ*, which balances how much to weight between *ab initio* and experimental loss. We find that some values of *λ* can improve small ring performance beyond even the baseline, although this comes at the cost of performance on the big rings. This tradeoff is explored more in Fig. 7.

## 4 Methods

Here we will provide more detailed explanations of our models, including how we generate features from molecules, how those features are transformed into predictions, and how we quantify uncertainty in those predictions. Recall the graphical model of a molecule, where atoms are vertices and bonds are edges. Then, our predictions correspond to two graph properties: per-vertex (shift) and per-edge (coupling). Different GNN layer structures can be used to update different graph properties towards the goal of making predictions on those properties. Here we detail the two layer types which we can employ in parallel or separately to generate predictions. We refer to these layers as the message passing layers, which update only the per-vertex features in the graph, and decode layers, which update both the vertex and edge features using a dense *N* × *N* representation of all possible edges in a graph. In this section, we discuss our methodology for representing a molecule as a graph, using our GNN layers to make predictions, and improving predictions through better use of data and quantifying uncertainty in predictions.

### 4.1 Featurization

Featurization is the process of converting a molecule into a set of feature matrices that can be used for the learning task. We begin by converting a molecule to a graph, an intuitive process given our typical model of a molecule. We can think of each atom as a vertex and each bond as an edge. Information about the molecular graph, as well as its geometric structure, are turned into relevant feature matrices in three steps.

In the first step, we create per-vertex features using basic properties of atoms. For each atom, we generate a tensor which includes the atomic number, the default and total valence of the atom, partial and formal charge of the atom, and other atom specific properties. These properties are all obtained directly from the RDKit^22^ Molecule object and the periodic table. They are collected into a tensor that we will refer to as *x*, which has shape *N* × *f*_v_, for *N* atoms in the molecule and *f*_v_ per-vertex features.

In the second step, we create a set of adjacency matrices directly from the graph. There are five total adjacency matrices, each of shape *N* × *N* and each symmetric. The first has a 1 wherever two vertices have any bond between them and 0s everywhere else. The remaining only place 1s where there is a specific bond type between the two vertices, with one adjacency matrix for each of single, double, triple, and bond order 1.5. This set of adjacency matrices is collected into a tensor that we will refer to as *G*_adj_ which has shape *N* × *N* × 5.

Finally, we create a set of per-vertex-pair properties using geometric properties of the molecule. Specifically, we examine the distances between pairs of atoms and the angles between atoms which share a neighbor. These properties are calculated from an ensemble of molecular conformers that specify the 3D structure (position of each atom) of the molecule, allowing us to easily compute distances and angles between pairs of atoms. In experimental NMR, conformational variability leads to the observation of a weighted average of the parameters of each possible conformer, so we aim to capture more than a single value for the distance and angle features. We include the (Boltzmann weighted) mean over the conformers for some features, but also use a collection of Gaussians to parameterize the distribution over the conformers more thoroughly. We collect these features as *G*_feat_, which has a shape *N* × *N* × *f*_e_, for *f*_e_ per-vertex-pair features.

**Fig. 8:**
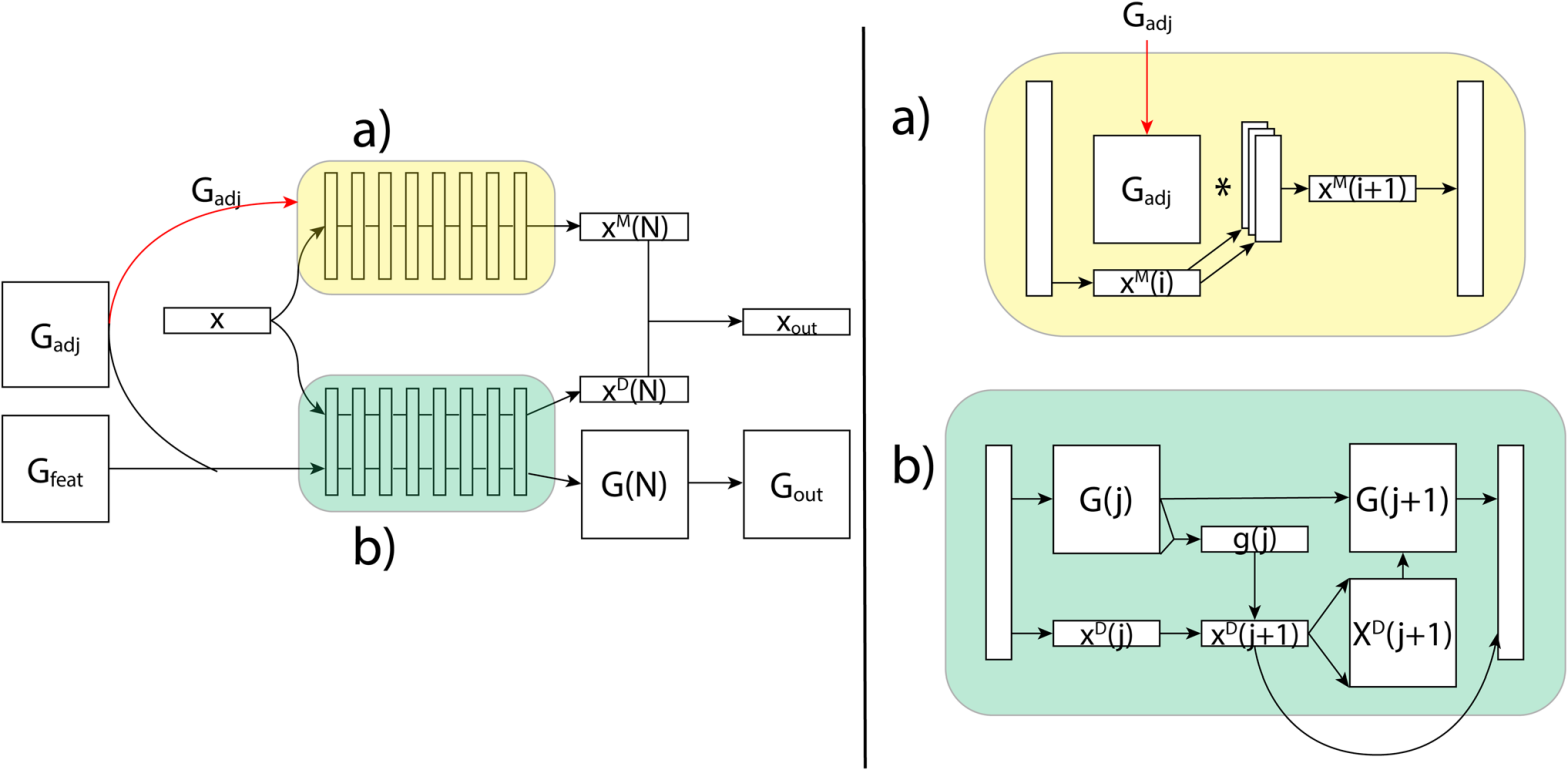
An illustration of the FullSSPrUCe model. Inputs *x*, *G*_adj_ and *G*_feat_ are generated from the molecule and its conformers, and then fed into the message passing (a) and decode (b) layers. The final outputs are used to make chemical shift (*x*_out_) and scalar coupling (*G*_out_) predictions.

Generating these conformers has thus far been the slowest part of the prediction pipeline, but has shown promise in increasing accuracy and provides the only features that are capable of distinguishing stereoisomers. As a proof of concept, we started out using an ensemble of conformers drawn from the Boltzmann distribution through parallel tempering (PT). These PT conformers provide us with highly accurate distances and angles as well as an accurate sampling of the distribution of conformers, which is important for capturing the conformational variability. However, we are aiming to do rapid predictions of NMR properties, so we turn to a conformer generation method which, while less accurate, is much faster. To that end, we use ETKDG,^11^ a conformation generation method which uses distance geometry and experimental torsion-angle preferences and other basic knowledge to create conformers. We then apply a single MMFF94 optimization step, which includes calculating the Boltzmann weight of each conformer, and that weight can be used for the averaging of the features from each conformer. Through the use of the ETKDG conformers and basic molecular properties, we are able to rapidly generate feature vectors for each atom and each atom-pair (see ESI† for full details of features used). These vectors are fed as the inputs *x*, *G*_adj_ and *G*_feat_ to the GNN, as shown Fig. 8.

### 4.2 Message passing layers

The GNN receives these three feature tensors and passes them to its two layers types. The simplest layer type in our GNNs are the message passing layers, which are a common method for molecular property prediction tasks.^23^ These layers update only the per-vertex properties using a simple aggregation scheme over each vertices' neighbors. The message passing layers take as inputs *x* and *G*_adj_, and do not consider *G*_feat_ at all. As shown in Fig. 8, the first step is to match the sizes of *G*_adj_ and *x*, which is done using an independent set of linear layers. Then, the message passing scheme updates the features in *x* through matrix multiplication with the adjacency matrix. This is collected back down to the correct size, where non-linearities and normalization can be performed. The final output, *x*_out_, has shape *N* × *f*_out_, where *f*_out_ can be adjusted using the initial linear layers.

The message passing scheme can be augmented to pass messages beyond a single step quite easily. To do so, we add layers to *G*_adj_ which correspond to powers of *G*_adj_. For example, *G*_adj_^3^ allows us to pass messages between vertices connected by three bonds. We can also add the identity matrix to *G*_adj_ to add self-loops. All of this can be done within the context of matrix multiplication and the stacking of our additional adjacency matrices to create layers with multiple types of message passing occurring simultaneously. Using this basic GNN message passing algorithm through matrix multiplication, these layers update per-vertex properties in a manner that can be useful for predicting shift values, but does not incorporate geometric properties efficiently.

### 4.3 Decode layers

Our second layer type, decode layers, incorporate all features and can predict scalar coupling by alternating between updating per-vertex and per-edge properties. As shown in Fig. 8, we now use *x*, *G*_adj_ and *G*_feat_. We start by stacking *G*_adj_ and *G*_feat_ into a single tensor *G*, which has shape *N* × *N* × (5 + *f*_e_), so that it represents 5 + *f*_e_ features for each possible edge. Each decode layer proceeds in two steps by first updating the per-vertex properties, then updating the per-edge properties. Each update is done using a gated recurrent unit (GRU) which takes in the original values of the property to update (the hidden state) and an input to use to calculate an update. It then calculates an update and a weighting and outputs the weighted sum of the hidden state with the update.

In the decode layers, we begin by updating per-vertex properties by using *x* as the hidden state and *G* as the input. *G* needs to be reshaped, which is done using an aggregation function over one of the *N* dimensions. This results in the update *x*_out_, which is then expanded to be used as the input to the second GRU, whose hidden state is the original *G*. This creates the update *G*_out_, which, along with *x*_out_, can then be passed through a non-linear layer and normalization before being passed to the next layer.

This repeated updating structure allows us to use the geometric features more naturally by updating them progressively using the relevant atomic features. Since both *x* and *G* are updated, we can use these outputs to predict both shift and coupling values. However, even in scenarios where we are only predicting shifts, these layers can be valuable in using the geometric features, and so we combine the updated per-vertex features from decode and message passing layers into a single per-vertex tensor that is then subjected to final layers for shift predictions. Through this combination of layers, we can consider atomic and interatomic features to produce full spin system predictions.

### 4.4 Uncertainty quantification

While machine learning models do a great job of fitting data and making predictions, they tend to be difficult to understand, especially when dealing with deep models. Most crucially, we know that they will always have some error, so understanding whether a given prediction is more or less accurate than others can be very useful. To that end, we took efforts to quantify the uncertainty in our predictions. We have ultimately settled on a form of ensembling that we will refer to as bootstrapping, since it is derived from bootstrap ensembling.^24^ Ensembling is the process of generating a prediction or decision from the results of a set (or ensemble) of individual predictors, sometimes called inducers.^25^ Commonly, ensembles are used because the majority prediction or average prediction from the set of inducers can be more accurate than any one inducer.^25^ Ensemble techniques can also be used to generate measures of uncertainty in predictions based on the variance in the predictions of the inducers^24^ or more complex measures on this set of predictions.^26^ Uncertainty quantification may also be accomplished in deep learning through generative models for density estimation or other Bayesian methods,^27^ however ensemble-based techniques have shown promise in chemical prediction tasks,^28^ including NMR-related tasks,^9,29^ so we base our uncertainty quantification on these ideas. This ensemble based, variance uncertainty may be somewhat restrictive, being primarily useful for uncertainty regarding the coverage of chemical space. However, its capacity to learn multiple “motifs” in its separate inducers may be particularly useful in chemical prediction tasks, where repeated functional groups are of interest.

**Fig. 9:**
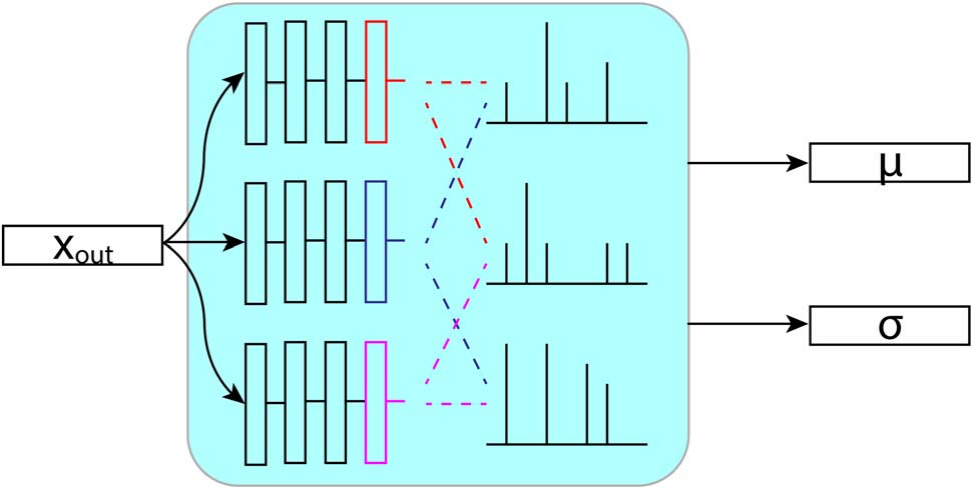
Each layer sees a different subset of the data at train time. Then, at test time, we use the mean and standard deviation across the layers to make predictions and quantify uncertainty.

Our bootstrapping method, as shown in Fig. 9, creates an ensemble of multiple FullSSPrUCe models, where each share most of their parameters. This differs from most ensemble methods where each inducer is trained completely separately, sometimes with entirely different types of models.^25^ Ours differ only on their final layers (those after the message passing and decode layers), which are simple residual and linear layers directly modifying *x*_out_ and *G*_out_, which we call the bootstraps. At train time, the error for a given molecule is decided by considering the predictions from only a subset of the bootstraps, by taking the mean over that subset. This allows each bootstrap to learn from a different subset of the training data without duplicating the training of the shared parameters.

At test time, we generate our predictions using the mean of all of the models' predictions, but we can examine their differences in predictions to quantify the uncertainty. We use the standard deviation of the models' individual predictions as the uncertainty in our ensembled prediction, which has proven to correlate quite strongly with accuracy. Through our bootstrapping method, we are provided with a quantification of the uncertainty in a prediction, an important step towards better understanding and using the model.

### 4.5 Learning jointly between experimental and *ab initio*

We are also interested in training for two types of data: *ab initio* and experimental. *Ab initio* data is generated through simulation, and so tends to be cheaper than experimental data to obtain. Thus, it has been used in most previous ML prediction tasks, where large amounts of data are needed to generate good models. Further, experimental data tends to be noisy and is susceptible to human error, making it more difficult to predict. But, our goal is ultimately to predict experimental results, so we must find a way to use all the data we have in an intelligent way.

The naive way to use all our data would be to simply augment our experimental training set with as much *ab initio* data as we can obtain. The main issue is that *ab initio* data is known to have systemic errors, and so we need to make some distinction between *ab initio* and experimental. The classic ML method for doing so is to use transfer learning, typically by pretraining a model on *ab initio* data and then ‘fine-tuning’ it on the experimental data. Guan *et al.* use this method with their GNN to moderate success. However, this assumes that experimental values are always the gold standard for our training data, which we know they are not. Misassigned peaks, missing values, and experimental noise can degrade this data.

Our approach seeks to learn from *ab initio* and experimental data jointly, as in the naive approach, while favoring the experimental values, as in the transfer learning approach, but with an awareness of the flaws experimental data presents. We do so using a technique termed disagreement regularization, which compares *ab initio* and experimental values for a given molecule to decide how to assign the loss. Specifically, we create two output ‘channels’, one which seeks to predict the *ab initio* values for a molecule and one which seeks to predict experimental values. Creating these channels is as simple as having the final linear layers produce a result which is *N* × 2 rather than *N* × 1 for shift predictions. When we have only experimental or only *ab initio* data for a particular atom, we simply set the loss for that value as the loss along the appropriate channel. For the remaining values, the loss function is then a weighted combination of the loss on these two channels, where the weight is determined by how much the *ab initio* and experimental ground truths agree.

How should we choose the weight between our two channels? Our observation is that when *ab initio* and experimental data agree, both are likely to be good. When both are good, we are more interested in predicting experimental values. When the data disagree, it is most likely a result of noise or error in the experimental data, since *ab initio* data is relatively noise-free and does not suffer from human error. So, when the agreement is strong, we should weight towards experimental data, and when agreement is weak, we should weight towards *ab initio*. This is reflected in the loss function in eqn (1), where *L*_a_ and *L*_e_ are the losses on the *ab initio* and experimental channels, respectively, *ϕ*_a_ and *ϕ*_e_ are the *ab initio* and experimental values given in the data, and *λ* is a tunable hyperparameter. We have chosen to leave the 1 ppm offset in the denominator fixed, but future work may explore the effects of adjusting this value as well.1
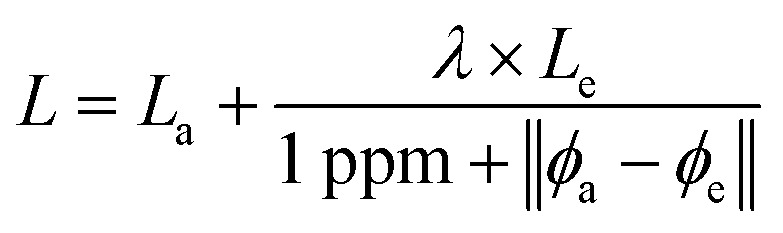


Our hope is that this loss function will combat problems in both data sources. Because the two channels share inputs and parameters up to the very final layer, we believe that this loss function encourages the model to learn the difference between *ab initio* and experimental data to correct the systematic error in *ab initio* data. By weighting down the loss when experimental data is likely to be incorrect, as identified by its disagreement with *ab initio* data, the model can ignore potentially noisy or erroneous data. With the main goal of predicting experimental results, our disagreement regularization loss intelligently combines *ab initio* and experimental data.

## 5 Discussion

FullSSPrUCe is a neural network based model for full spin system NMR parameter prediction. We built on previous graph neural network approaches to learn shift and coupling parameters, while also incorporating molecular geometry to be able to distinguish stereoisomers and improve performance. We introduced a novel method for incorporating experimental and *ab initio* in training.

As shown in Section 3.1, our model has lower errors on its testing set than do the models to which we compared. Each previous model has made its own different choices, and we believe that our combination of choices has led to this improved performance. IMPRESSION is the only non-GNN based method. The Jonas and Kuhn GNN is the only method to use exclusively topological information. CASCADE intentionally restricted its dataset, particularly for experimental data, due to the potential unreliability of NMRShiftDB data, and did not train any models exclusively on experimental data. Unlike all previous methods, we do not draw features from a single conformer, but rather from an ensemble of conformers, which seems to makes the features more useful. We combine the largest amount of data with the most consistently successful aspects of each previous model, and as a result we have seen the best performance. This performance is a strong step forwards, but there are remaining areas that deserve further exploration.

One such area is solvent and temperature effects, which we ignore because user-contributed NMRShiftDB^17^ spectra often do not have solvent or temperature labels, which may contribute to error in multiple ways. Primarily, it will increase noise through unaccounted experimental variability. We suspect it also contributes to a noticeable difference between error rates for protons bonded to carbons and those bonded to other atoms. Across models, we see much better accuracy on predictions of ^1^H shifts for protons bonded to carbons, while those bonded to nitrogens, oxygens and other heavy atoms have significantly higher error (full details in ESI†). This is consistent with *ab initio* results, which tend to be further from experimental values for protons not bonded to carbons. These effects can often be corrected by accounting for solvent and temperature,^30^ which provides an interesting avenue of future work.

Recall that we use ETKDG conformers to create geometric features of molecules as inputs to our network. We showed a tradeoff between accuracy and number of conformers, which serves as a proxy for time spent generating conformers. This tradeoff is certainly not exclusive to our methods, as all computational techniques make some trade off between theory and time (*e.g.* molecular dynamics *vs.* distance geometry, *etc.*). Even other neural network models have this trade off.^10^ However, since our model takes particular metrics of the distribution of properties across conformers, we believe that we could improve on the tradeoff by calculating these metrics directly, leveraging machine learning techniques rather than generating a set of conformers to measure the metrics. We would also like to improve on the stereoisomer identification task that relies on these conformers. Whether this improvement is through faster, more accurate geometry features or better training of molecules with stereoisomers, we believe that we can achieve DFT level accuracy in this task.

Our disagreement regularization tool should also continue to be developed, as it provides us with an interesting method for combining experimental and *ab initio* data. We believe that the inclusion of the full *ab initio* dataset allows the model to learn the differences between *ab initio* and experimental values, and thus correct its small ring predictions accordingly. The noiseless *ab initio* data may also allow us to correct errors in experimental data, especially misassignments of spectra to their correct atoms, allowing us to surpass our baseline in some cases. However, the effects of certain hyperparameters present some unusual results and need further study to be fully understood.

## 6 Conclusion

Through the use of graph neural networks, we are able to predict per-vertex and per-edge properties corresponding to NMR parameters, specifically chemical shift and scalar coupling values, respectively. We achieve state of the art performance on experimental and *ab initio* prediction tasks, as well as providing a novel method for training on both datasets. Through the incorporation of molecular geometry, we are able to be useful for downstream tasks such as stereoisomer identification. Lastly, our uncertainty measure allows us to be much more confident in the accuracy of a large subset of our predictions, while filtering out those that are likely to have large error.

## Data availability

Code is available on GitHub at https://github.com/thejonaslab/fullsspruce-public.

## Author contributions

E. J. conceptualized the work. J. L. W. and E. J. designed the work, acquired data, analyzed data, developed code used in this work, and wrote and edited the manuscript.

## Conflicts of interest

There are no conflicts to declare.

## Supplementary Material

SC-014-D3SC01930F-s001
