# Supplementary material for: Rapid prediction of full spin systems using uncertainty-aware machine learning
Source: Chem Sci. 2023 Sep 15;14(39):10902–13. doi: 10.1039/d3sc01930f (PMC10566464; doi:10.1039/d3sc01930f)
Supplement: SC-014-D3SC01930F-s001 [file SC-014-D3SC01930F-s001.pdf]

Electronic Supplementary Material (ESI) for Chemical Science.
This journal is © The Royal Society of Chemistry 2023

Supplementary Information for Rapid Prediction of Full Spin Systems using Uncertainty-Aware Machine Learning

Jake Williams, Eric Jonas

Contents

1 Data **2**
1.1 NMRShiftDB 2
1.2 GDB-17 4
1.3 Ab Initio Data 6

2 Methods **8**
2.1 Featurization 8
2.1.1 Atoms 8
2.1.2 Atom Pairs 9
2.1.3 ETKDG 9
2.2 Hyperparameter Selection 9
2.3 Train Test Split 10

3 Additional Results **11**
3.1 Full Coupling Results 11
3.2 BMRB Shifts 14
3.3 Quantified Uncertainty 16
3.4 Effects of Ab Initio Errors 19
3.5 Full DP4 Results 22
3.6 Multi Shift Models 23
3.7 Training Set Size 24

4 Ab Initio Simulation **25**

1 Data

1.1 NMRShiftDB

The majority of experiments run were trained and evaluated using data from the NMRShiftDB[1]. We selected molecules with at most 128 atoms (including protons) and with only atoms H, C, O, N, F, S, P and Cl. Below are histograms showing the distribution of observed experimental shifts of the protons and carbons for our selected molecules from NMRShiftDB, as well as the distribution of the size of the selected molecules in terms of number of atoms.

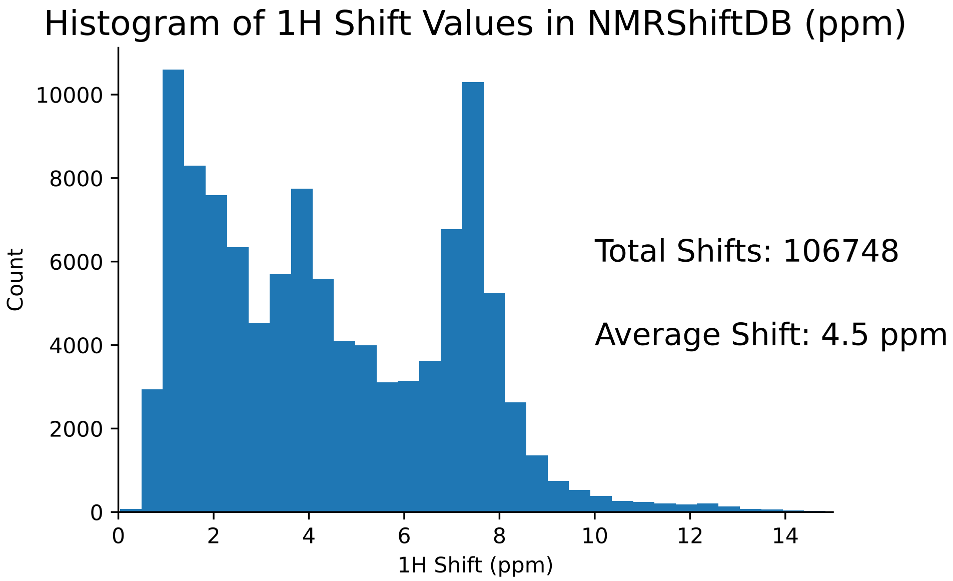

Figure 1: Distribution of measured experimental shifts for all protons in NMRShiftDB in ppm.

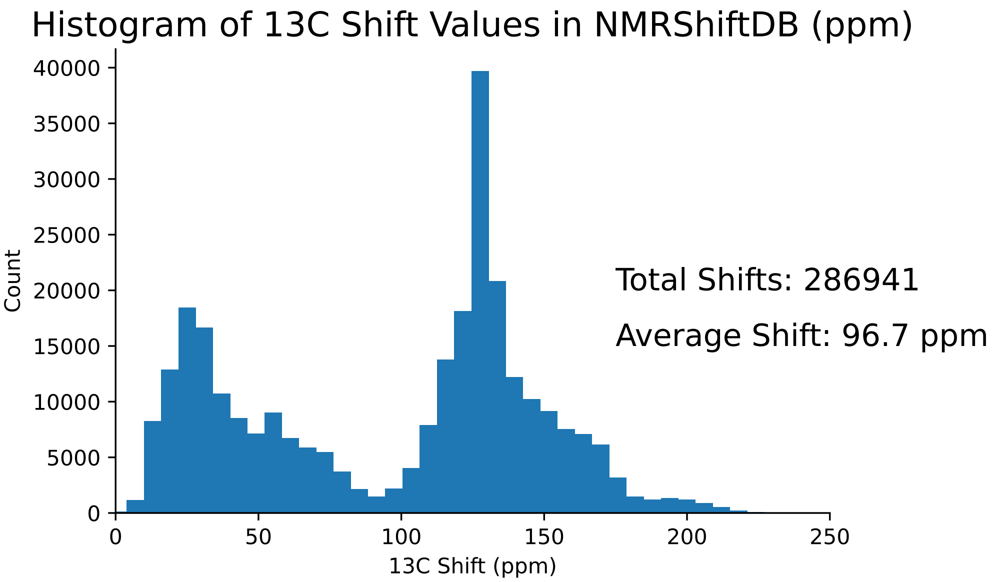

Figure 2: Distribution of measured experimental shifts for all carbons in NMR-ShiftDB in ppm.

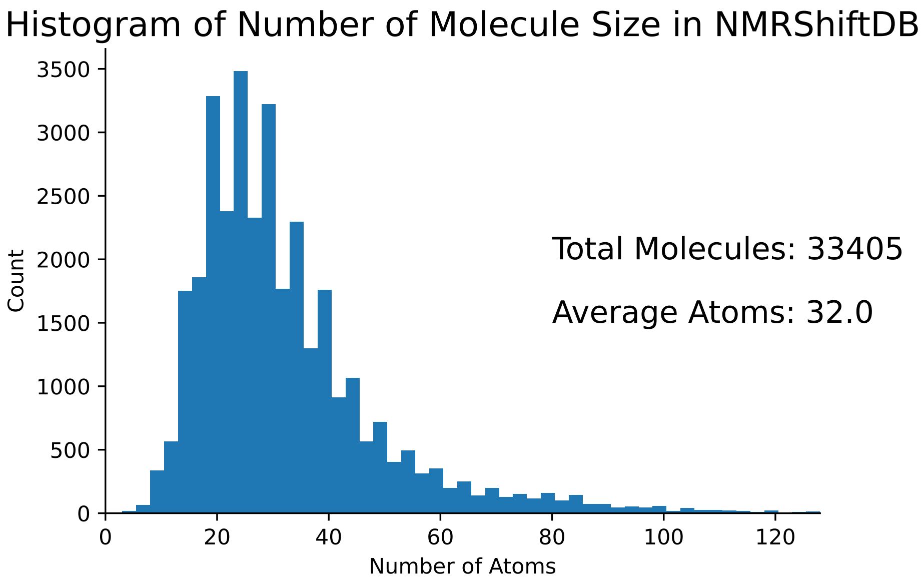

Figure 3: Distribution of number of atoms in the molecules for each molecule in NMRShiftDB.

1.2 GDB-17

For experiments studying the effects of stereoisomers, we used data from GDB-17[2], which contains multiple stereoisomers of the same molecules for some molecules. Due to the size of the entire GDB-17 dataset, we selected a more manageable subset to use in our experiments. We also applied the same size and atom restrictions as in our selections from NMRShiftDB. Below are histograms showing the distribution of observed shifts of the protons and carbons for our selected molecules from our subset of GDB-17 and the size of the molecules. From this GDB-17 subset, we created unique data for multiple stereoisomers of some molecules, and so also included is a histogram showing the distribution of the number of stereoisomers per molecule.

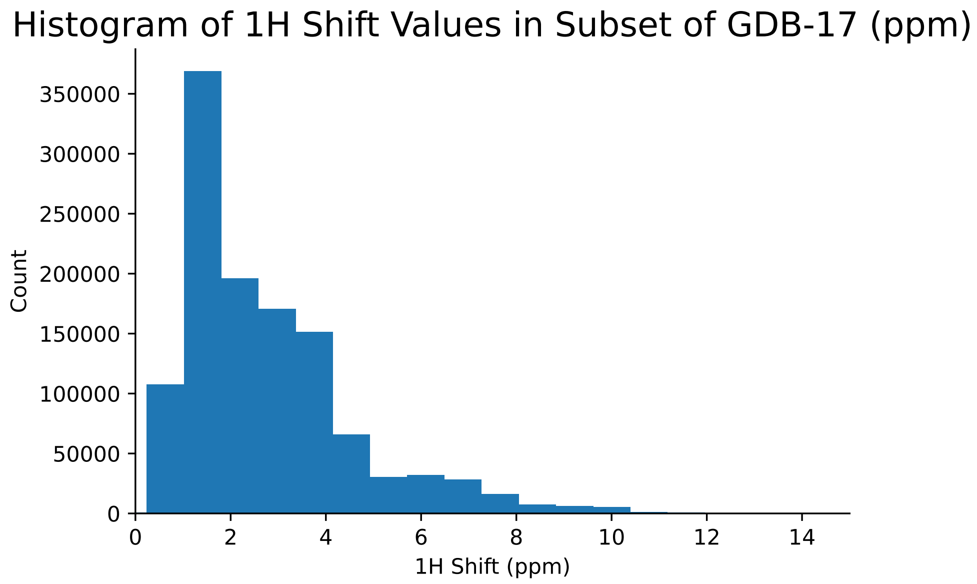

Figure 4: Distribution of calculated ab initio shifts for all protons in chosen subset of GDB-17 in ppm.

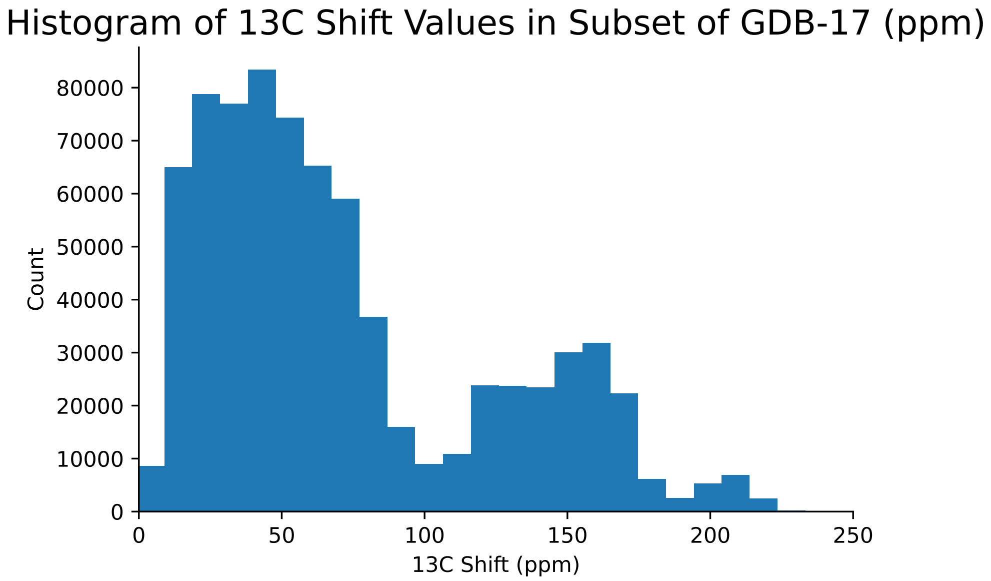

Figure 5: Distribution of calculated ab initio shifts for all carbons in chosen subset of GDB-17 in ppm.

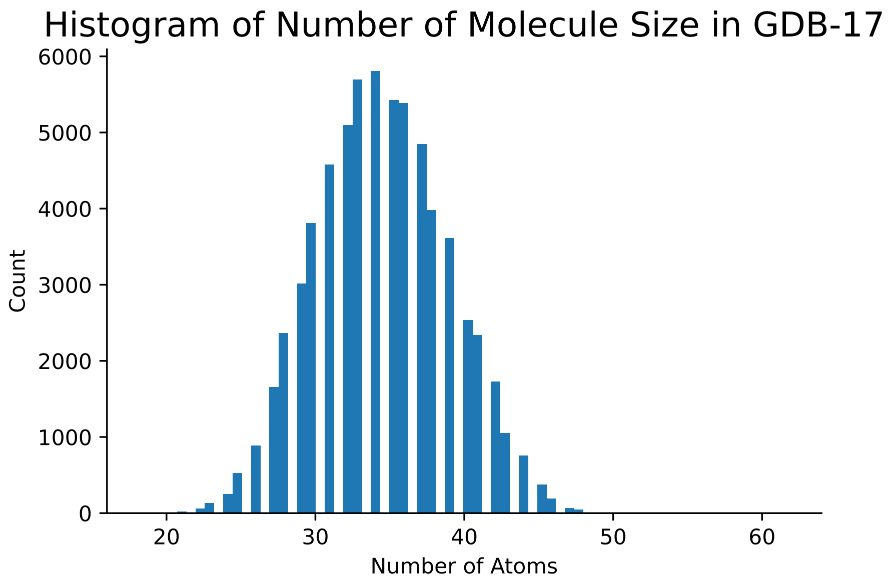

Figure 6: Distribution of number of atoms in the molecules for each molecule in selected GDB-17 subset.

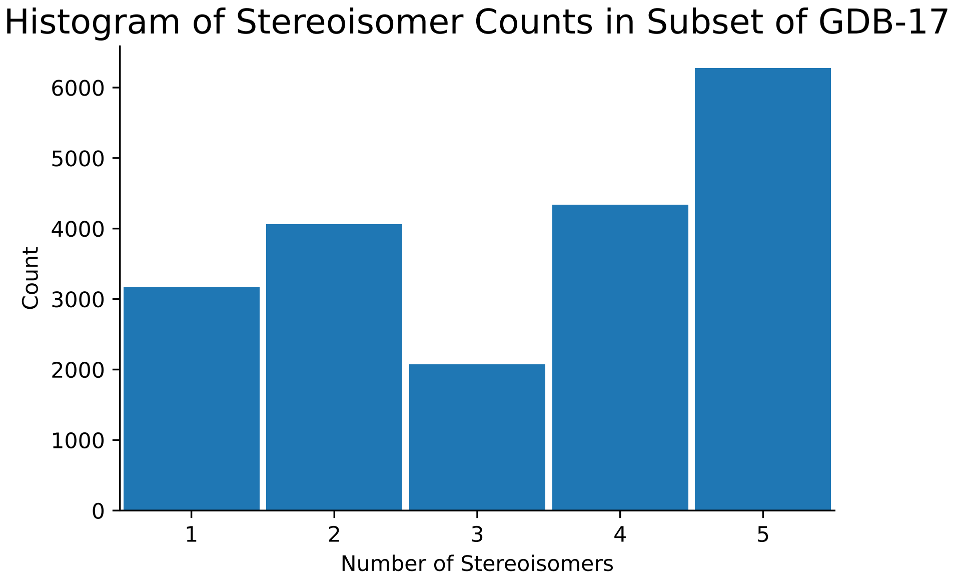

Figure 7: Distribution of number of stereoisomers per molecule for chosen subset of GDB-17.

1.3 Ab Initio Data

For each molecule in NMRShiftDB, we generated simulated results using DFT (for details see Section 4). In Figure 8, we compare these ab initio values on proton shifts to our experimental data. We note that ab initio data is imperfect, with a strong tendency to report smaller than experimental values. However, these errors to be most prevalent in protons which are not bonded to carbons,

which is seen easily when coloring the values accordingly. Figure 8 shows that the majority of ab initio errors are made on protons bonded to either nitrogens or oxygens (with other non-carbon options removed from the plot for simplicity). We examine the effect this has on our trained models in Section 3.4

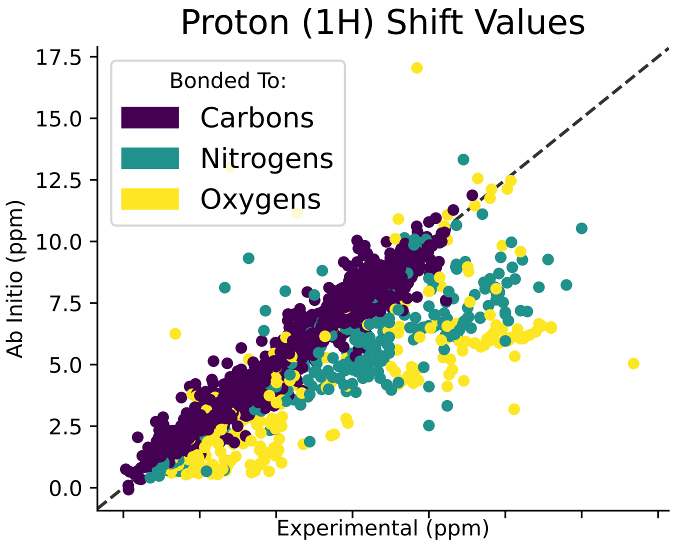

Figure 8: Ab initio data compared to experimental data for ^{1}H shift predictions, marked by the atom to which the proton is bonded. Ab initio is noticeably more reliable in its measurements of shifts for protons bonded to carbons.

2 Methods

2.1 Featurization

Below we describe how a molecule is featurized. This process generates feature vectors for each atom (collected in x) and for each pair of atoms (collected in symmetric matrices G_{adj} and G_{feat}). The tables below detail each element of these feature vectors.

2.1.1 Atoms

Table 1: Features per Atom

Feature	Description	Number of Elements
Atomic Number	One hot encoded from {H, C, O, N, F, P, S, Cl}	8
Valence	Int and one hot encoded from 1-6	7
Aromaticity	Whether atom is in aromatic structure, determined by RDKit	1
Hybridization	One hot encoded from $\{s, sp, sp^2, sp^3, sp^3d, sp^3d^2$, UNSPECIFIED$\}$	7
Formal Charge	Presence of net charge, one hot encoded from {-1, 0, 1}	3
Default Valence	Valence of atom on periodic table, one hot encoded from 1-6	6
Rings	Whether the atom is in a ring of size N for N from 3-8	6
Chirality**	One hot encoded from RDKit ChiralTypes	9
Electronegativity**	Fixed value according to atomic number	1
MMFF Atom Types**	Atom type from RDKit's MMFFMolProperties, one hot encoded	51
Total		99

**Coupling model only features. Note: RDKit ChiralTypes: {UNSPECIFIED, Tetrahedral CW, Tetrahedral CCW, OTHER, Tetrahedral, Allene, Square Planar, Trigonal Bipyramidal, Octahedral}. MMFF Atom Types selected: [1, 2, 3, 4, 5, 6, 7, 8, 9, 10, 11, 12, 15, 16, 17, 18, 20, 21, 22, 23, 24, 25, 26, 27, 28, 29, 30, 31, 32, 33, 37, 38, 39, 40, 42, 43, 44, 46, 48, 59, 62, 63, 64, 65, 66, 70, 71, 72, 74, 75, 78].

2.1.2 Atom Pairs

Table 2: Features per Atom Pair

	Feature	Description	Number of Elements
	Bonded	Do atoms share any bond	1
G_{adj}	Bond Type	One-hot encoding of bond type from [1,1.5,2,3] (or all zeros if no bond)	4
	Distance	$\min(d^{-n/4}, 2)$ for mean distance between atoms d and n from 4-39	36
G_{feat}	Conf Gauss	Average value of a Gaussian on distance given by set of conformers	20
	R Gauss	Set of Gaussians evaluated on mean distance d	26
	Angle Gauss	Set of Gaussians evaluated on mean angle a (in radians)	33
Total			120

Conf Gauss parameters: $\sigma = 0.2$, μ evenly chosen 20 times from 0.3-10. R Gauss parameters: $\sigma = 0.2$, $\mu = n/4$ for n from 2-27. Angle Gauss paramters: $\sigma = 0.1$ and μ from [0,1,3] and $\sigma = 0.01$ and μ from [1.70,1.72,1.74,1.76,1.78,1.80,1.82,1.84,1.87,1.89,1.91,1.93,1.95,1.97,1.99,2.01,2.03, 2.05,2.07,2.09,2.11,2.13,2.16,2.18,2.20,2.22,2.24,2.26,2.28,2.30]

2.1.3 ETKDG

ETKDG[3] is used to create the conformers from which the features above draw their distances and angles. This is done using RDKit's[4] EmbedMultipleConfs function, with maxAttempts set to 20. The molecule has all stereo and chiarlity tags set before being passed to the embedding function. If the embedding fails to generate the required number of conformers (50 throughout the paper), the molecule is considered invalid. The molecule is then run through MMFFOPtimizeMoleculeConfs to use RDKit's MMFF94 optimization on each conformer.

2.2 Hyperparameter Selection

The graph neural network architecture depends on the selection of a set of hyperparameters, or variables which are set for each experiment and not changed during training. Our model contains hyperparameters which control the number of layers, the size of hidden layers, the normalization functions used, the optimizer, learning rate and learning schedule used, dropout, as well as the number of bootstraps and the percentage of data each bootstrap sees. These hyperparameters were tested and modified over time based on empirical results to improve performance and avoid overfitting. For example, increasing the number of message passing layers improves performance up to between 6-8 layers, at which point overfitting reduces test set performance. Hyperparameters are not shared between models for predicting proton, carbon and coupling values.

2.3 Train Test Split

For each of our experiments, we split the datasets into train and test datasets. This was done using the Morgan fingerprint of each molecule, which can be converted into an integer[5]. The train/test split was then done by selecting molecules by the final digit of their fingerprint. In most experiments, the test molecules were those with final digits 0 or 1. This technique also allowed for easy cross-validation, which was done by averaging the error on five experiments, which used (0,1), (2,3), (4,5), (6,7) and (8,9) as their selections for the test set, respectively. Only the molecules in the train datasets were presented to the model during its training loops. All results presented throughout are based on the model's performance on test datasets.

3 Additional Results

3.1 Full Coupling Results

In Figure 9 we present scatterplots for all coupling types reported by our model. Different nuclei and longer range couplings are not included in the training data for our models, so our model does not report predictions for those coupling types. The choice of which coupling types to include was based on their relative importance and impact in expected use cases such as structure elucidation. In Figure 10, we present scatterplots for $^3J_{HH}$ coupling predictions for the small set of experimental coupling values referenced previously [6–8].

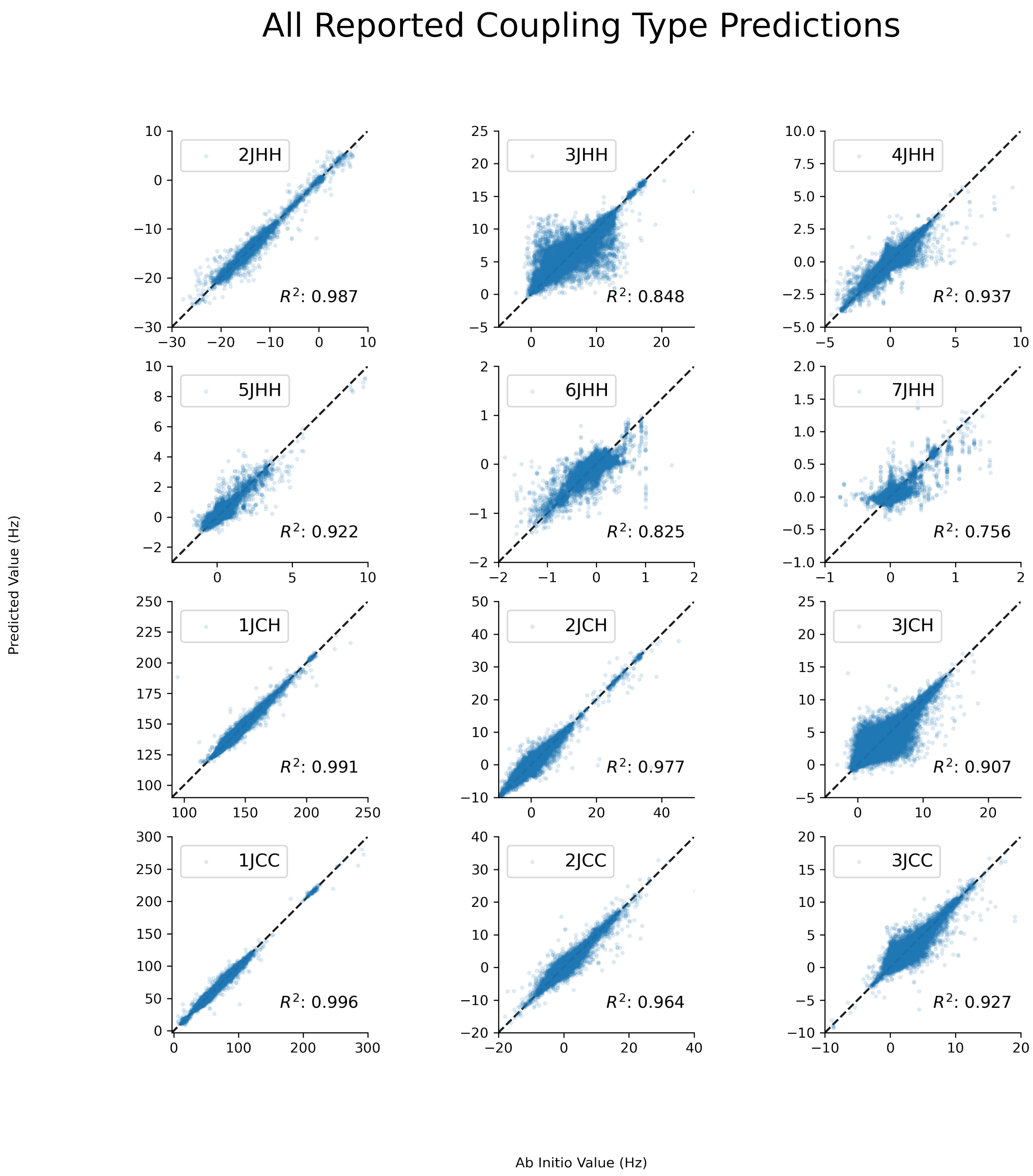

Figure 9: Comparison of predicted and ab initio scalar coupling values in Hz for all reported coupling types.

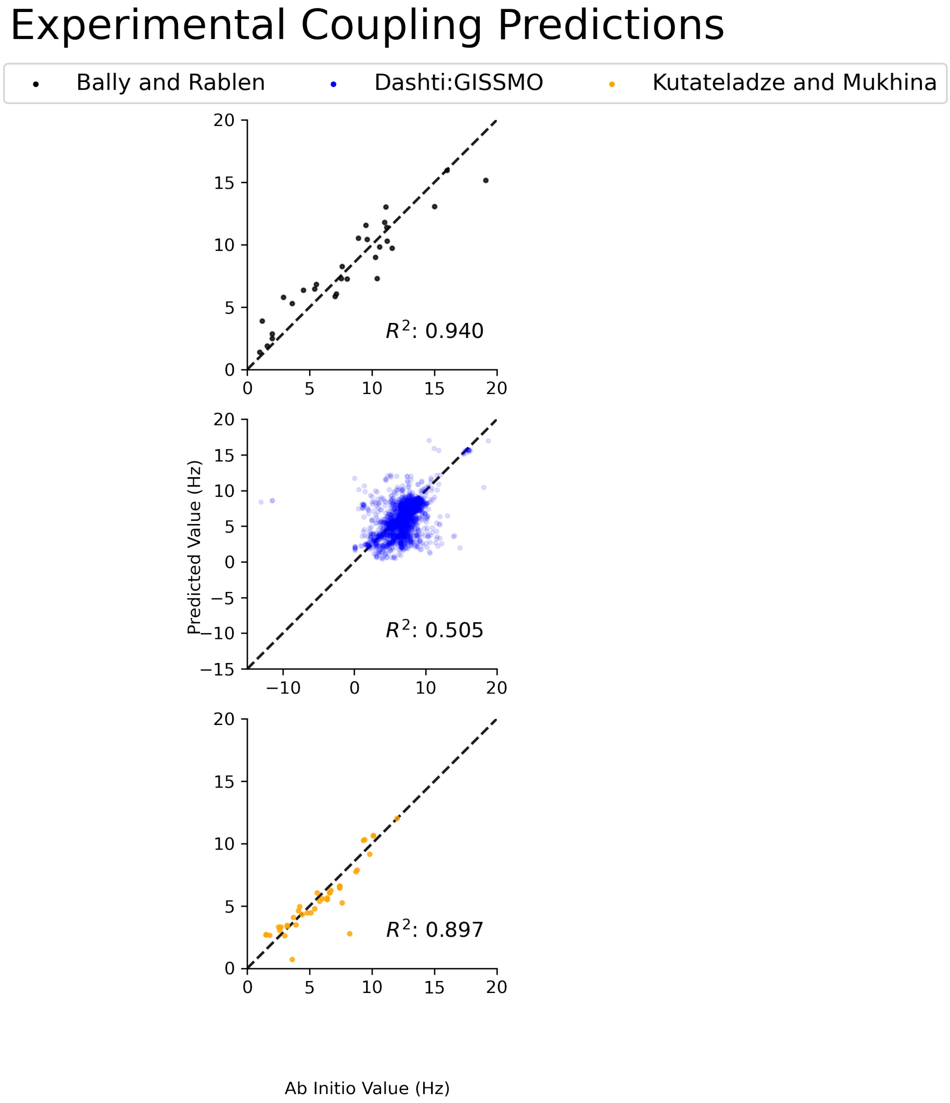

Figure 10: Comparison of predicted and experimental scalar coupling values in Hz for ${}^3_J HH$ coupling values on three small experimental datasets.

3.2 BMRB Shifts

With the exception of the small coupling datasets, all of our previous results have evaluated our models using the same datasets they were trained on, using the train/test splits as described earlier. Here, we will evaluate our default proton and carbon shift models on a small subset of data from the BMRB[9]. Figure 11 shows scatterplots of the experimental and predicted values for 100 molecules, chosen to ensure they were valid for our model and not in the training set. They can be found on `https://bmrb.io` in the metabolomics dataset, by the IDs listed below (i.e. ID 1 corresponds to bmse000001):

BMSE IDs: 1, 2, 3, 4, 5, 6, 7, 8, 10, 11, 12, 13, 15, 16, 17, 18, 19, 20, 22, 23, 25, 26, 27, 28, 29, 30, 31, 32, 33, 34, 35, 36, 37, 38, 39, 40, 41, 42, 43, 44, 45, 46, 47, 48, 49, 50, 51, 52, 53, 54, 55, 56, 57, 58, 59, 60, 61, 62, 63, 64, 65, 66, 67, 68, 69, 70, 71, 72, 73, 74, 75, 76, 77, 78, 79, 80, 83, 84, 86, 87, 89, 90, 91, 92, 93, 94, 95, 96, 97, 98, 99, 100, 101, 102, 103, 104, 105, 106, 107, 108

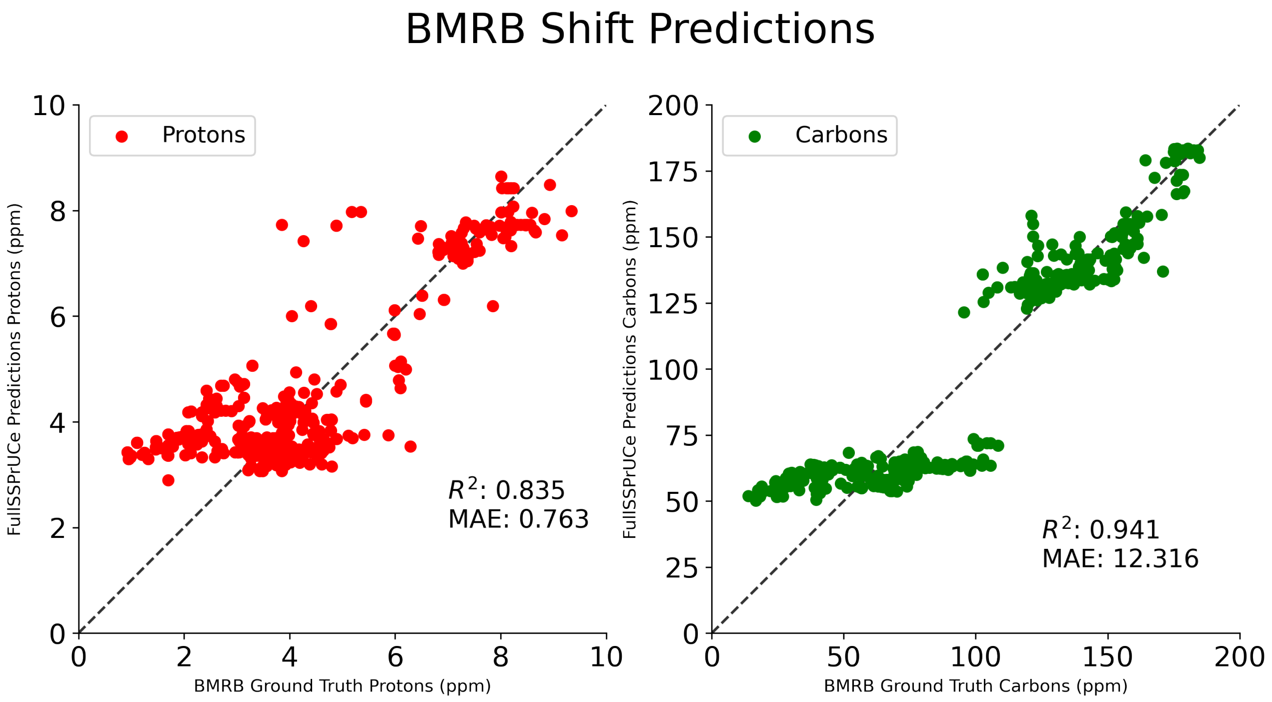

Figure 11: Comparison of predicted and experimental ^{1}H and ^{13}C shifts on 100 molecules from the BMRB[9].

3.3 Quantified Uncertainty

Here, we examine two more results regarding the uncertainty quantification provided by our model. The first is a closer look at the exact breakdown of errors when sorted according to the quantified uncertainty. Similar to the plots showing the mean and 95th percentile error, the x-axis in Figure 12 refers to the fraction of predictions with lower uncertainty. The y-axis is the error in the prediction (in Hz or ppm). This plot emphasizes the outliers in the error, especially when the error is much higher than others with similar uncertainties. It also once again demonstrates the difference between experimental and ab initio tasks' error distributions, with ab initio tasks having less variance in the error distributions.

Then, o further illustrate the effectiveness of our bootstrapping method for uncertainty quantification, we compare to a simpler method. In Figure 13, we once again present our ^{1}H shift predictions sorted according to the uncertainty value produced by our bootstrapping method. We also present these predictions sorted according to what percentage of the training data is near the predicted value. We call this the "Data Seen" sorting method. For the below figures, the exact value is derived by measuring what percentage of the training data is within 0.3 ppm of the predicted value. We expect that if our predicted value lies near many training data points, then our model should produce more accurate results. We observe a small correlation here, especially in the ab initio data, however the bootstrapping method far and away outperforms the Data Seen method, especially in identifying the very most accurate results.

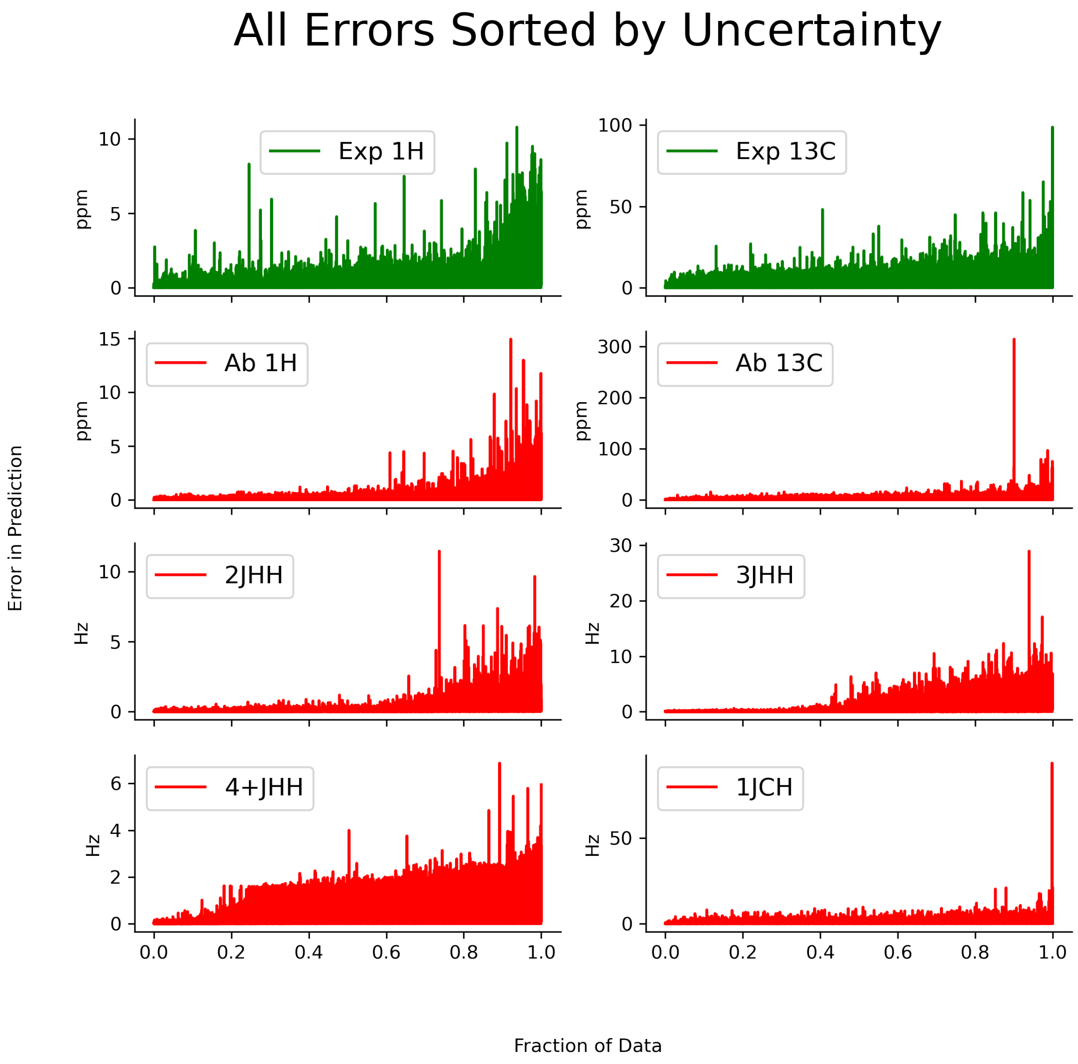

Figure 12: An Improvement Factor chart, similar to those in the DP4 paper [10], showing the improvement factor for each molecule, with correct structures in blue and decoys in green.

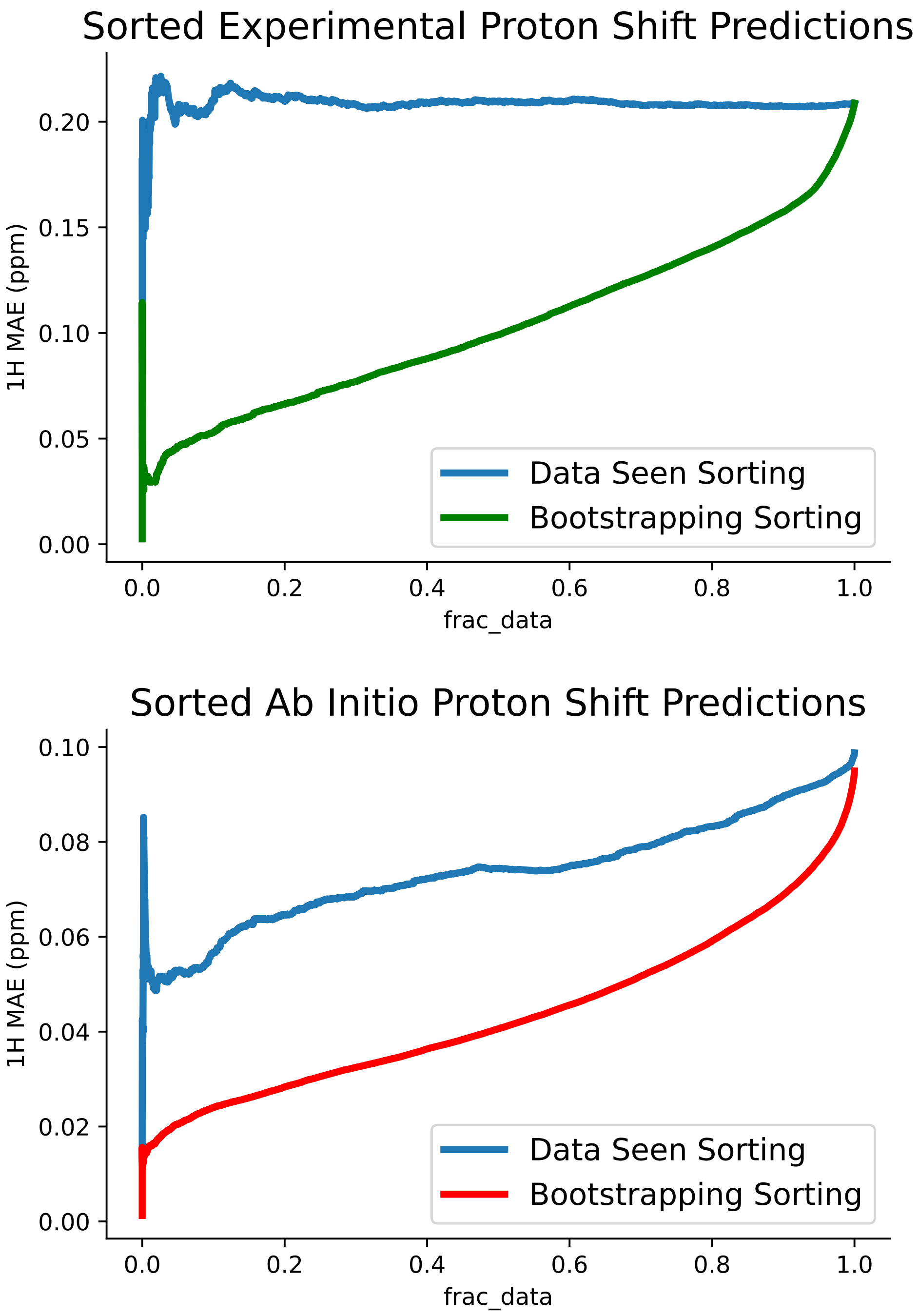

Figure 13: Rolling mean average error of predictions sorted using bootstrapping and a simple comparison to the training data set, on both experimental and ab initio prediction tasks. Our bootstrapping method produces uncertainty values that reliably correlate with accuracy far beyond what the simple method produces.

3.4 Effects of Ab Initio Errors

In Section 1.3, we saw that there are systematic errors in the ab initio data we generated, especially related to the identity of the molecule to which a proton is bonded. Here, we examine whether this effects persists in the models we build. In Figure 14, we look at four models' predictions of proton shift values compared to the experimental values, similarly to Figure ??. Our default model, in the top left of Figure 14, is trained only on experimental data, so it does not learn any noticeable systematic errors. However, when we train a model using ab initio data, we learn the systematic errors that are present in that data. The bottom two models were parts of the disagreement regularization experiments, where we use both ab initio and experimental data to train a model. In the ab initio baseline model, we added in ab initio data for the unobserved small ring molecules, but do not treat this data differently. In the disagreement regularization model, we add in all of the ab initio data and then predict two channels for the two data types. Both of these models perform similarly in Figure 14, however, we can see differences when we move to Figure 15.

In Figure 15, we look only at the predictions made on the small ring molecules, which is a much smaller dataset, and one for which none of the models shown were provided experimental training data. This includes the top left plot, which is now the experimental control, which was trained on only big ring experimental data. In the top right and bottom left, we can see that the systematic error persists for both models that were provided ab initio training data but were not trained using disagreement regularization. However, we see that this is corrected by the disagreement regularization model in the bottom right. This gives us hope that the disagreement regularization model is capable of learning about systematic differences between the different datasets it was trained on.

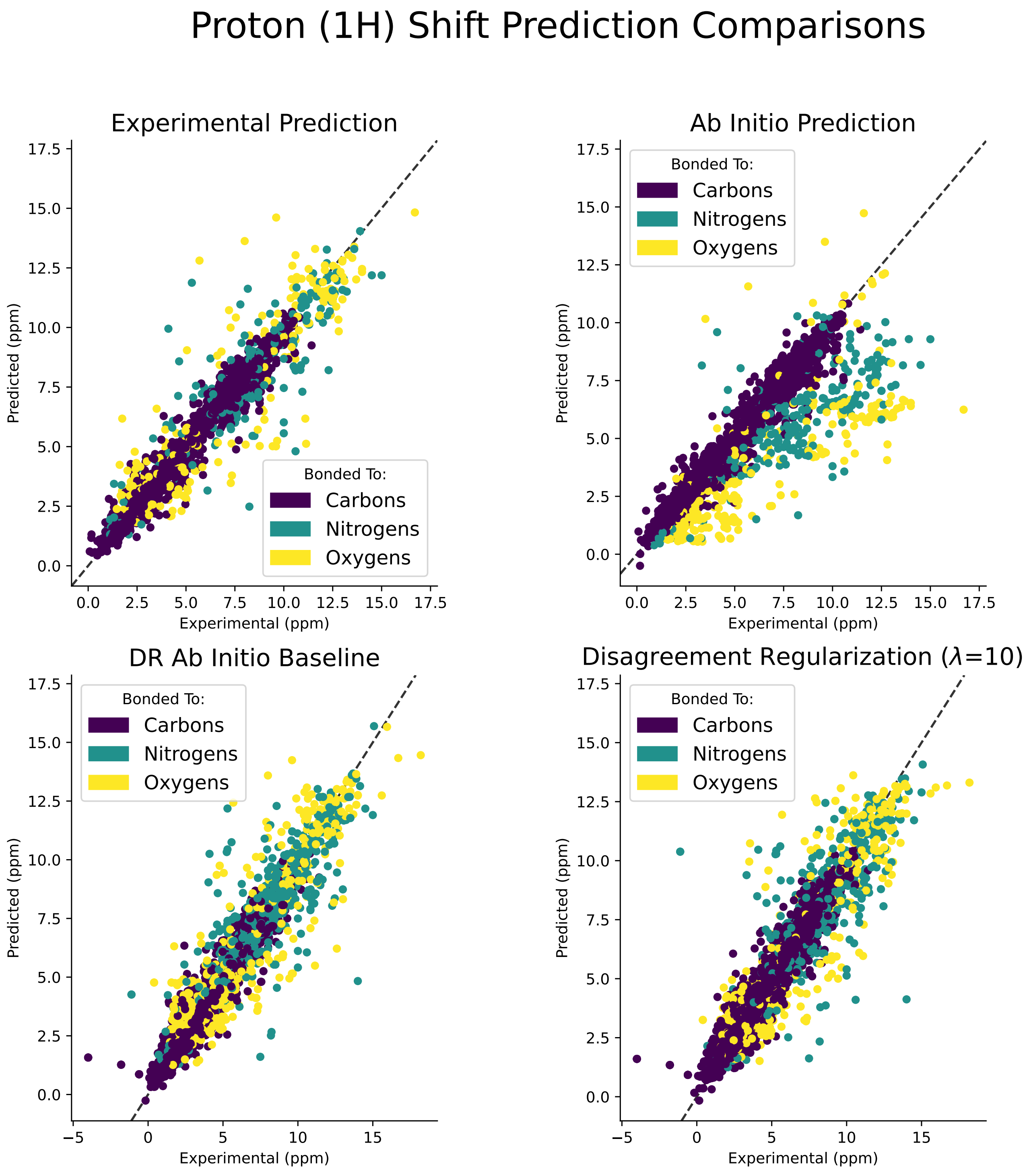

Figure 14: Predictions from different models compared to experimental data for ^{1}H shift predictions, marked by the atom to which the proton is bonded.

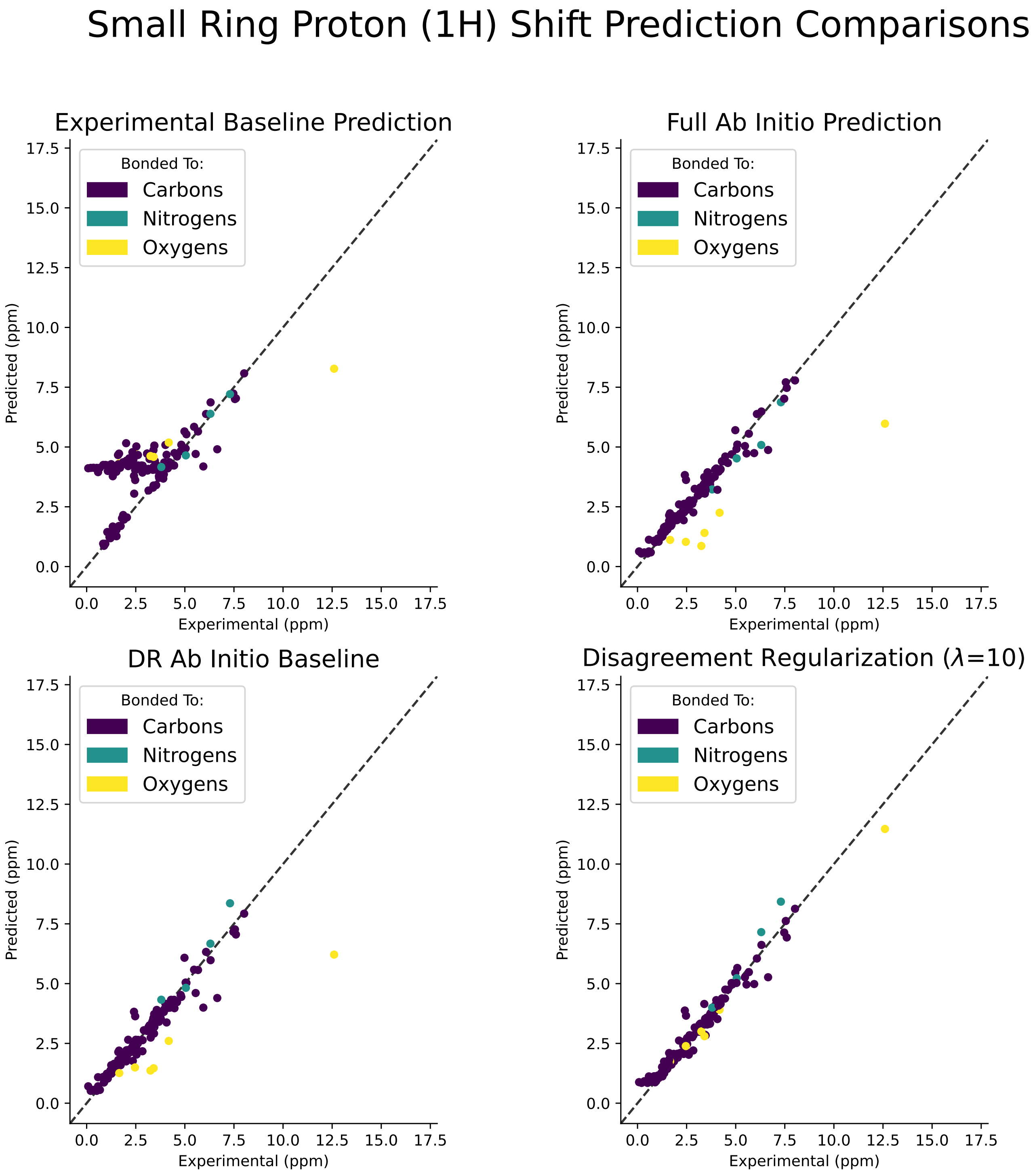

Figure 15: Predictions from different models compared to experimental data for ^{1}H shifts in small ring molecules, marked by the atom to which the proton is bonded.

3.5 Full DP4 Results

As noted in the main text, our calculations are less reliable in finding the correct structure from the candidates than the original DP4 work was. We found the correct structure 45% of the time for those molecules with all 8 candidate structures, which is clearly better than random guessing, but not the most informative measure. To see how those predictions break down, we have charted the improvement factor for each candidate structure in Figure 16. The improvement factor was introduced in the original DP4 paper [10], and is equal to the probability assigned to a structure times the number of candidate structures, as this gives the ratio between our assigned probability and a uniformly assigned probability. In the chart, the improvement factor for correct structures are plotted in blue, which we would like to be as high as possible, with the incorrect structures plotted in green. Note that the cutoffs we used are slightly different than in the original paper, because we have a maximum of 8 structures, so there is a maximum improvement factor of 8. Figure 16 reinforces that our model often does very well, with about half of incorrect structures having improvement below 0.1, and over half of correct structures having improvement greater than 1. There are still outliers in the correct and incorrect structures, but the overall shape of the chart is promising.

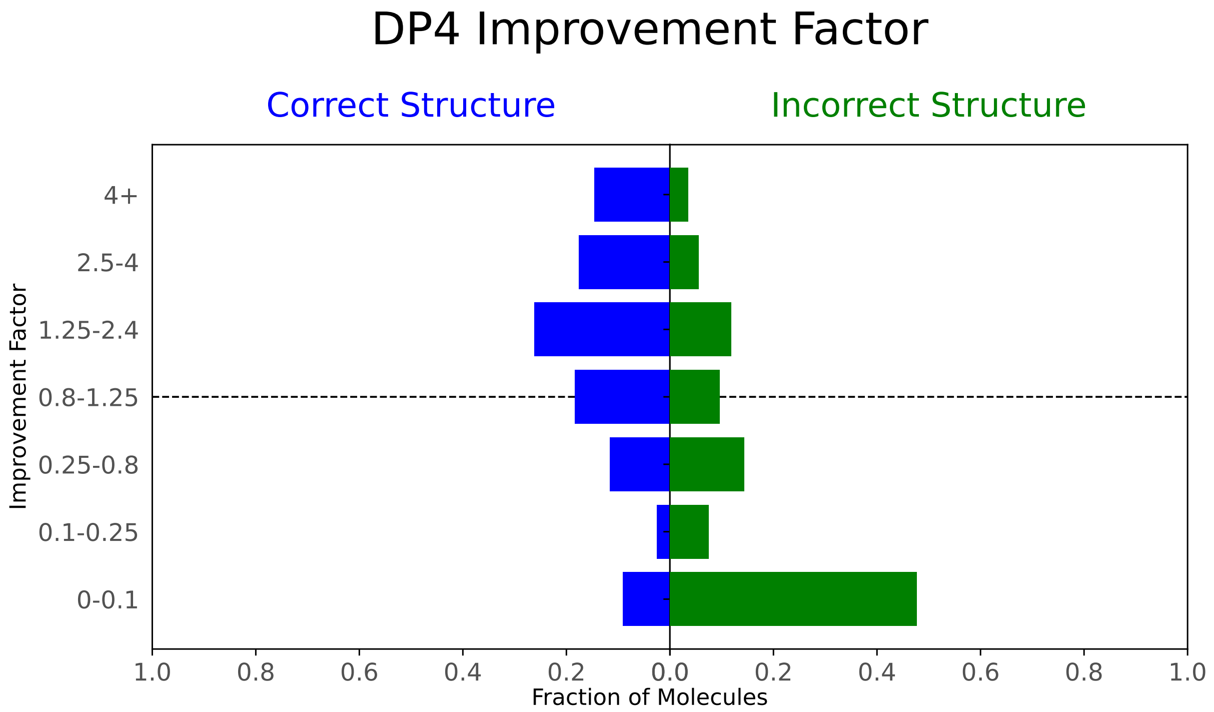

Figure 16: An Improvement Factor chart, similar to those in the DP4 paper [10], showing the improvement factor for each molecule, with correct structures in blue and decoys in green.

3.6 Multi Shift Models

Our model is designed to use three separate neural networks to predict ^{1}H, ^{13}C and coupling values. However, it can predict all three simultaneously. Doing so tends to reduce performance, however, so our default is to use models trained separately on only one of the three. In Figure 17, we compare four models trained to for simultaneous ^{1}H and ^{13}C prediction (multi shift models). These models have an extra hyperparameter, HL, which weights the losses for these two types of predictions. Figure 17 demonstrates that increasing HL improves performance on ^{1}H shifts at the cost of performance on ^{13}C shifts, and vice versa, however we lose performance on both compared to the individual models.

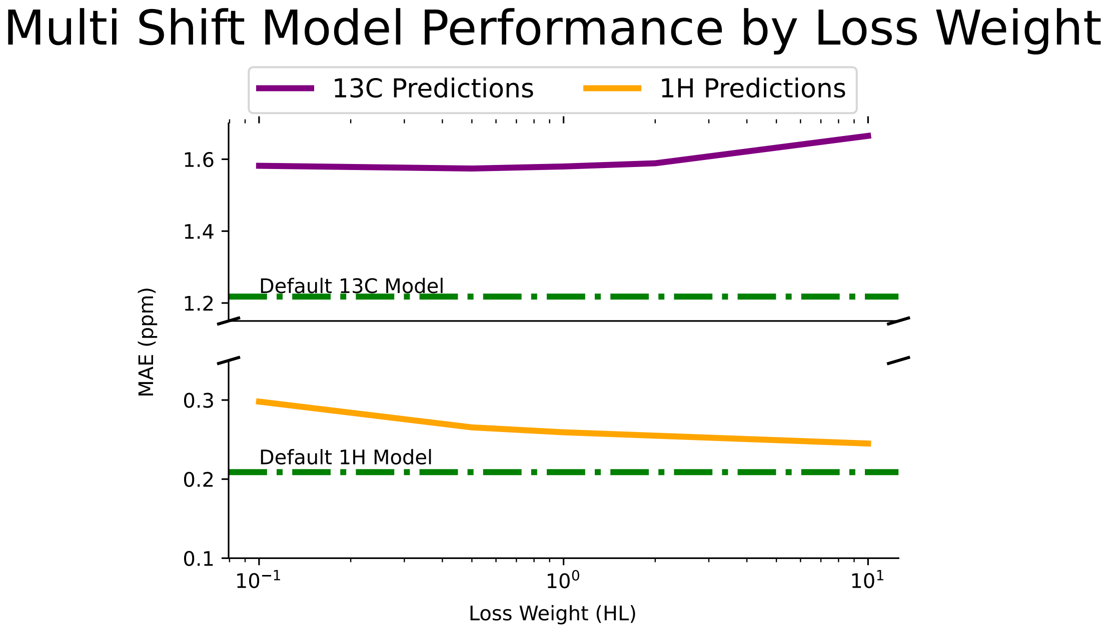

Figure 17: Performance of Multi Shift Models with different weights. Weighting the loss function trades off performance between the two types of shifts, but performance degrades compared to the two separate models.

3.7 Training Set Size

We also examined the effect of the total quantity of training data by training five additional models with varying amounts of ^{1}H training data. Note that all models were tested on identical ^{1}H testing sets, yielding the results in Figure 18. More training data unsurprisingly improves performance, but it is encouraging to see that the uncertainty quantification is as valuable with less training data. We also see a plateau of the improvement in performance, which holds up even for the top 10 and top 50 percent most confident predictions.

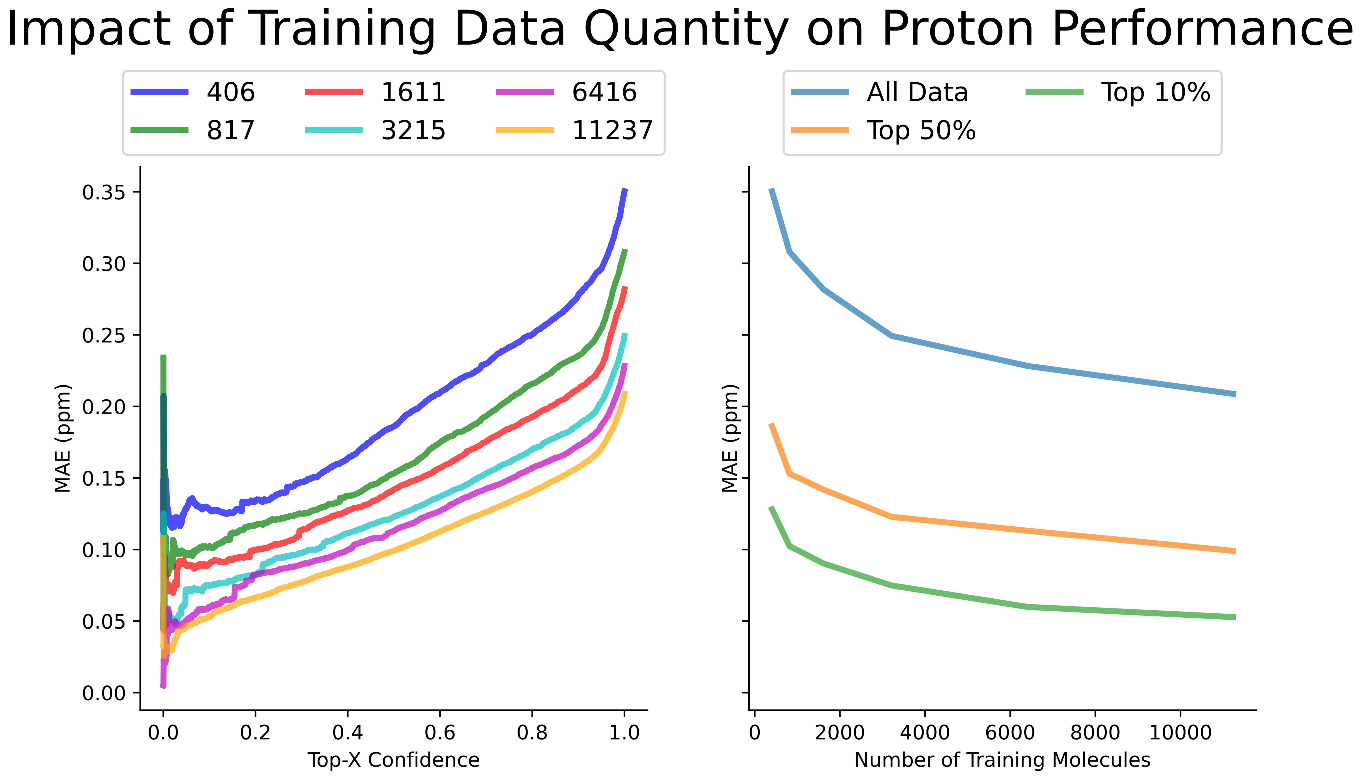

Figure 18: Performance of Multi Shift Models with different weights. Weighting the loss function trades off performance between the two types of shifts, but performance degrades compared to the two separate models.

4 Ab Initio Simulation

Ab initio simulations were performed by identifying low-energy conformers generated from parallel tempering and performing gas-phase geometry optimization using the Orca software package at the B3LYP/6-31g level of theory. Isotropic shielding and coupling constants were then calculated via GIAO (again with Orca) at B3LYP/6-311g using a implicit (SMD) chloroform solvent model and Boltzmann-weighted with the final DFT energies. Shields were converted to shifts via standard linear scaling procedures referenced to a small experimental dataset.

References

[1] S. Kuhn and N. E. Schlörer, *Magnetic Resonance in Chemistry*, 2015, **53**, 582–589.

[2] L. Ruddigkeit, R. van Deursen, L. C. Blum and J.-L. Reymond, *Journal of Chemical Information and Modeling*, 2012, **52**, 2864–2875.

[3] S. Riniker and G. A. Landrum, *Journal of Chemical Information and Modeling*, 2015, **55**, 2562–2574.

[4] G. Landrum, *RDKit: Open-source Cheminformatics*, 2006.

[5] D. Rogers and M. Hahn, *Journal of Chemical Information and Modeling*, 2010, **50**, 742–754.

[6] T. Bally and P. R. Rablen, *The Journal of Organic Chemistry*, 2011, **76**, 4818–4830.

[7] H. Dashti, W. M. Westler, M. Tonelli, J. R. Wedell, J. L. Markley and H. R. Eghbalnia, *Analytical Chemistry*, 2017, **89**, 12201–12208.

[8] A. G. Kutateladze and O. A. Mukhina, *The Journal of Organic Chemistry*, 2015, **80**, 5218–5225.

[9] J. C. Hoch, K. Baskaran, H. Burr, J. Chin, H. R. Eghbalnia, T. Fujiwara, M. R. Gryk, T. Iwata, C. Kojima, G. Kurisu, D. Maziuk, Y. Miyanoiri, J. R. Wedell, C. Wilburn, H. Yao and M. Yokochi, *Nucleic Acids Research*, 2022, **51**, D368–D376.

[10] S. G. Smith and J. M. Goodman, *Journal of the American Chemical Society*, 2010, **132**, 12946–12959.
